# Cooperative and acute inhibition by multiple C-terminal motifs of L-type Ca^2+^ channels

**DOI:** 10.7554/eLife.21989

**Published:** 2017-01-06

**Authors:** Nan Liu, Yaxiong Yang, Lin Ge, Min Liu, Henry M Colecraft, Xiaodong Liu

**Affiliations:** 1 X-Lab for Transmembrane Signaling Research, Department of Biomedical Engineering, School of Medicine, Tsinghua University, Beijing, China; 2 Department of Physiology and Cellular Biophysics, Columbia University, New York, United States; 3 School of Life Sciences, Tsinghua University, Beijing, China; 4 IDG/McGovern Institute for Brain Research, Tsinghua University, Beijing, China; National Institutes of Health, United States

**Keywords:** L-type Ca2+ channels, Ca2+ channel inhibition, calmodulin, distal carboxyl terminus, Ca2+ dependent inactivation, None

## Abstract

Inhibitions and antagonists of L-type Ca^2+^ channels are important to both research and therapeutics. Here, we report C-terminus mediated inhibition (CMI) for Ca_V_1.3 that multiple motifs coordinate to tune down Ca^2+^ current and Ca^2+^ influx toward the lower limits determined by end-stage CDI (Ca^2+^-dependent inactivation). Among IQ_V_ (preIQ_3_-IQ domain), PCRD and DCRD (proximal or distal C-terminal regulatory domain), spatial closeness of any two modules, *e.g.*, by constitutive fusion, facilitates the trio to form the complex, compete against calmodulin, and alter the gating. Acute CMI by rapamycin-inducible heterodimerization helps reconcile the concurrent activation/inactivation attenuations to ensure Ca^2+^ influx is reduced, in that Ca^2+^ current activated by depolarization is potently (~65%) inhibited at the peak (full activation), but not later on (end-stage inactivation, ~300 ms). Meanwhile, CMI provides a new paradigm to develop Ca_V_1 inhibitors, the therapeutic potential of which is implied by computational modeling of Ca_V_1.3 dysregulations related to Parkinson’s disease.

**DOI:** http://dx.doi.org/10.7554/eLife.21989.001

## eLife digest

All cells need calcium ions to stay healthy, but having too many calcium ions can interfere with important processes in the cell and cause severe problems. Proteins known as calcium channels on the cell surface allow calcium ions to flow into the cell from the surrounding environment. Cells carefully control the opening and closing of these channels to prevent too many calcium ions entering the cell at once. Ca_V_1.3 channels are a type of calcium channel that are important for the heart and brain to work properly. Defects in Ca_V_1.3 channels can lead to irregular heart rhythms and neurodegenerative diseases such as Parkinson’s disease.

Studies have shown that part of the Ca_V_1.3 channel that sits inside the cell – known as the “tail” – responds to increases in the levels of calcium ions inside the cell by closing the channel. The tail region of Ca_V_1.3 contains three modules, but how these modules work together to regulate channel activity is not clear.

Liu, Yang et al. investigated whether the three modules need to be physically connected to each other in the channel protein. For the experiments, several versions of the protein were constructed with different combinations of tail modules being directly linked as part of the same molecule or present as separate molecules. When any two modules were directly linked, the third module could bind to them and this was enough to close the Ca_V_1.3 channel. However, the channel did not close if the modules were totally isolated from each other as three separate molecules.

Certain types of neurons in the brain produce electrical signals in a rhythmic fashion that depends on Ca_V_1.3 channels. In Parkinson’s disease, increased movement of calcium ions into these neurons via Ca_V_1.3 channels interferes with the rhythms of the signals and can cause these cells to die. Liu, Yang et al. performed computer simulations to analyse the effects of closing Ca_V_1.3 channels in these neurons. The results suggest that this can restore normal rhythms of electrical activity and prevent these cells from dying.

The next step is to understand the molecular details of how the tail region closes Ca_V_1.3 channels and its role in healthy and diseased cells. This may lead to new ways to block Ca_V_1.3 channels in different types of diseases.

**DOI:** http://dx.doi.org/10.7554/eLife.21989.002

## Introduction

L-type Ca^2+^ channels (LTCC or Ca_V_1 channels) play pivotal roles in numerous physiological functions by mediating Ca^2+^ influx and membrane excitability (Striessnig et al., 2014). Among four isoforms of Ca_V_1.1–Ca_V_1.4 in LTCC family, Ca_V_1.3 channels exhibit unique biophysical properties (Lieb et al., 2014; Xu and Lipscombe, 2001) involving diverse regulatory mechanisms (Ben-Johny and Yue, 2014; Christel and Lee, 2012; Huang et al., 2012; Tan et al., 2011). Widely distributed in various excitable tissues, including both cardiovascular and nervous systems (Brandt et al., 2003; Mangoni et al., 2003; Nemzou et al., 2006; Striessnig, 2007), Ca_V_1.3 is also involved in a wide spectrum of pathology, including multiple inheritable diseases (Pinggera and Striessnig, 2016; Platzer et al., 2000). As one prominent exemplar of its pathophysiological linkage, Ca_V_1.3 expressed in substantia nigra pars compacta (SNc) neurons tightly controls the autonomous subthreshold Ca^2+^ oscillations which have been considered to underlie the pathophysiology of Parkinson’s disease (PD) (Chan et al., 2007; Guzman et al., 2009, 2010).

Ca_V_1.3 is tuned by its own distal carboxyl tail (DCT) to compete with apoCaM (Ca^2+^-free calmodulin), which is pre-associated with the carboxyl terminus of the channel at preIQ_3_-IQ domain (denoted as IQ_V_). Closely involved in both apoCaM and Ca^2+^/CaM binding (Jurado et al., 1999), IQ_V_ plays important roles in channel functions (Ben Johny et al., 2013; Liu et al., 2010; Singh et al., 2006; Wahl-Schott et al., 2006). The competitive tuning is highly regulated by fluctuations of [apoCaM], the strength of particular DCT isoforms, or the apoCaM affinity with IQ_V_ (Adams et al., 2014; Bazzazi et al., 2013; Liu et al., 2010). DCT consists of two putative α-helical domains—a proximal and a distal C-terminal regulatory domain (termed PCRD and DCRD, respectively) (Hulme et al., 2006). Positively charged PCRD could coordinate with negatively charged DCRD, in the context of Ca_V_1.3 (Liu et al., 2010), to regulate effective CaM affinity to the IQ_V_ region of the channel. Besides the reported inhibition on Ca^2+^-dependent inactivation (CDI) (Liu et al., 2010; Singh et al., 2006; Wahl-Schott et al., 2006), several studies suggest that DCT also concurrently attenuates voltage-gated activation (VGA), reducing the maximum open probability and positively shifting the voltage dependence (Hulme et al., 2006; Lieb et al., 2014; Liu et al., 2016; Scharinger et al., 2015; Singh et al., 2008; Wahl-Schott et al., 2006). Altogether, this competitive tuning of Ca_V_1 gating (activation/inactivation) emerges as a new modality distinct from conventional inhibitions such as intensively-studied Ca^2+^ channel blockers (CCBs) that normally only reduce VGA (Hockerman et al., 1997), or Ca^2+^/CaM-triggered conformational changes that induce CDI (Ben-Johny and Yue, 2014). However, several key matters still remain to be clarified before such new CMI (C-terminus Mediated Inhibition) could be fully established. First, PCRD, DCRD and IQ_V_ seemingly work cooperatively to mediate CMI as suggested by aforementioned analyses, but the exact interrelationships among these three motifs are yet to be elucidated. Second, CMI is supposed to act on channels in an acute manner similar to widely-applied CCBs or like interventions, but direct evidence is still lacking. Third, CMI is expected to reduce the overall Ca^2+^ influx; however, it is still unclear whether and how CMI is able to ensure the actual inhibition when both VGA and CDI are attenuated, apparently leading to contradictory effects on Ca^2+^ influx.

Embarking on these frontiers regarding CMI, we unveiled the principle of cooperation among the three key C-terminal motifs, with which we developed chemical-inducible CMI for Ca_V_1.3 channels. We then resolved all the aforementioned questions crucial to establish CMI, and gained new insights into Ca_V_1.3 gating by comparing CMI and CDI. In addition, with computational modeling of Ca^2+^ oscillations and pacemaking behaviors under the control of Ca_V_1.3 channels in SNc neurons, we explored the potentials of CMI-based inhibitors as therapeutic interventions for Ca_V_1.3-related PD.

## Results

### Provisional CMI concurrently attenuates both CDI and VGA

**Figure 1.**
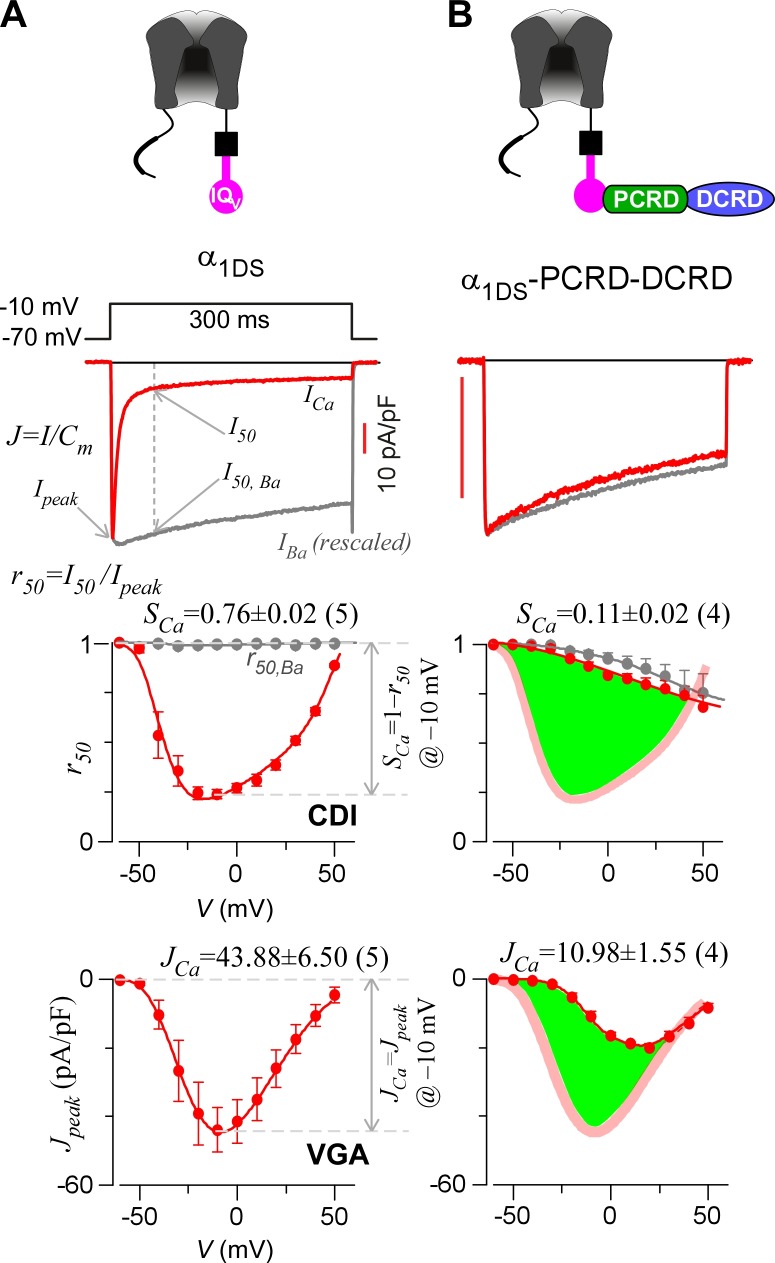
Inhibition of Ca_V_1.3 gating by carboxyl terminal motifs. (**A**) Parameters and indices illustrated by the control group of short Ca_V_1.3 channels. Representative current exemplars (Ca^2+^ current *I_Ca_* in red, with the scale bar in red; Ba^2+^ current *I_Ba_* in gray, rescaled) were shown for α_1DS_ at the membrane potential (*V*) of −10 mV, with the amplitudes measured at the time of peak (*I_peak_*) and 50 ms (*I_50_*). Inactivation profiles across the full range of *V* for *I_Ba_* and *I_Ca_* were quantified by the remaining current at 50 ms (*r_50_*), in ratio between *I_50_* and *I_peak_*. The CDI strength was quantified by 1−*r_50,Ca_* or *S_Ca_*, serving as one of the major indices. Based on Ca^2+^ current density normlized to the cell capacitance (*C_m_*), VGA in Ca^2+^ was profiled by *J_peak_* (in pA/pF), with the *J_peak_* amplitude at −10 mV or *J_Ca_* as the other major index. (**B**) In contrast to α_1DS_ channels with pronounced CDI (*S_Ca_*) and strong VGA (*J_Ca_*), α_1DS_-PCRD-DCRD incorporating all the three motifs of IQ_V_, PCRD and DCRD exhibited strong inhibitions on both CDI and VGA (less pronounced U-shape or V-shape), indexed with *S_Ca_* and *J_Ca_* (smaller values) respectively. Thick semi-transparent lines in red depict the CDI and VGA profiles of the α_1DS_ control group (**A**); and the differences in profiles (green areas as visual cues) illustrate the potency of CMI. **DOI:** http://dx.doi.org/10.7554/eLife.21989.003

Alternative splicing at the carboxyl terminus of α_1D_ results into two important native variants of long and short forms: α_1DL_ (exon 42) and α_1DS_ (exon 42A). Short variant of α_1DS_ terminates shortly after the IQ_V_ motif and lacks almost entire DCT domain, and its Ca^2+^ current (*I_Ca_*) exhibited strong CDI and VGA (Figure 1A). Regarding CDI, α_1DS_ still contains all the core elements including the IQ_V_ domain for apoCaM pre-association and the EF-hand motifs and N-terminal Ca^2+^/CaM binding NSCaTE motif (Dick et al., 2008) for CDI transduction. *I_Ca_* upon depolarization rapidly decayed (second row, red trace), indicative of strong CDI. In contrast, Ba^2+^ current (*I_Ba_*) decayed rather slowly (trace in grey) representing the background voltage-dependent inactivation (VDI). Thus, the fraction of peak current (*I_peak_*) remaining after depolarization for 50 ms (third row, *r_50_*) is closely correlated with inactivation, with the difference between the *r_50_* profiles in Ba^2+^ and Ca^2+^ serving as the ideal index of CDI. In practice, due to rather weak VDI of Ca_V_1.3 at 50 ms (*r_50,Ba_* close to 1) (Tadross et al., 2010), thus for simplicity the value of 1−*r_50,Ca_* at −10 mV was taken as the index of CDI strength (*S_Ca_*). VGA was evaluated by peak current density *J_peak_* (*pA*/*pF*), measured at different voltages while excluding potential artifacts due to cell-size factor (capacitance). Throughout this study, *S_Ca_* and *J_Ca_* (*J_peak_* at −10 mV) served as the quantitative indices for CDI and VGA respectively. Thus, by way of DCT competition against apoCaM, the provisional CMI was expected to attenuate both *S_Ca_* and *J_Ca_*, as suggested by prior studies (Liu et al., 2010; Singh et al., 2008, 2006; Tan et al., 2011, 2012; Wahl-Schott et al., 2006). However, most of these reports focused on either CDI or VGA only; or even when both were studied for whole-cell *I_Ca_*, CDI and VGA were separately evaluated with different experimental groups. Here, we conducted a side-by-side analysis for each variant or condition, not only more confirmative since concurrent changes in CDI and VGA would be mutually supportive, but also critical to later insights into CMI mechanisms and significance originated from such concurrency. Technically, compared to VGA and *J_Ca_*, CDI and *S_Ca_* are more robust so more favorable since CDI of Ca_V_1.3 is independent of global Ca^2+^ so insensitive to Ca^2+^-buffer capacities and variations in channel expressions partly due to transient transfections (Dick et al., 2008; Tadross et al., 2008). In general, for data and analyses we provided for both CDI and VGA, CDI (*S_Ca_*) was considered as the major index for CMI evaluations and VGA as the supplement.

**Figure 1—figure supplement 1.**
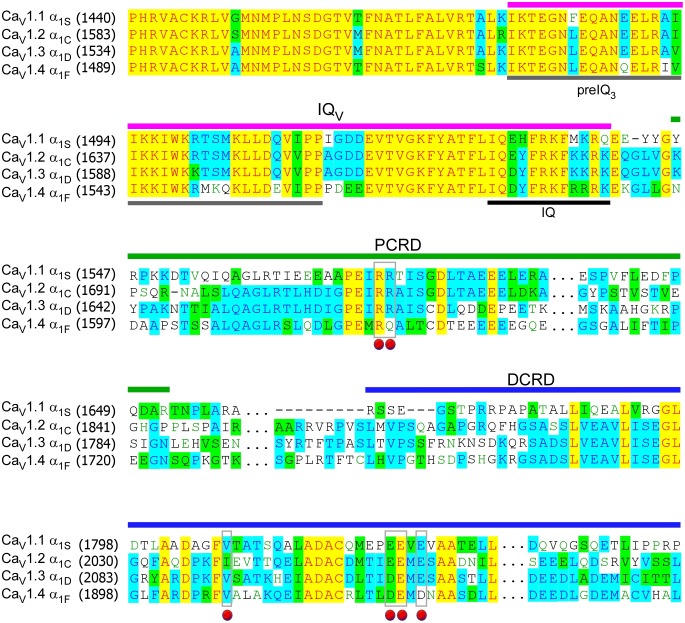
Sequence alignment of carboxyl termini for L-type Ca_V_1 channels. The sequences of α_1_ subunits were aligned for Ca_V_1.1 (α_1S_, XM_983862.1), Ca_V_1.2 (α_1C_, NM_199460.3), Ca_V_1.3 (α_1D_, NM_000720) and Ca_V_1.4 (α_1F_, NP005174), with GenBank accession numbers in parentheses. Key domains of pre-IQ_3_, IQ, IQ_V_, PCRD, and DCRD were underlined with different colors, and the conjunction domain (**…**) between PCRD and DCRD was skipped for clarity. Identical (yellow and blue), similar (green) and gapped (−) sequences were indicated. Key residues for potential interactions were marked by red dots, including valine (V) on DCRD, and residues of positively charged arginine (R) or negatively charged aspartic acid (D) or glutamine (E) on PCRD or DCRD, respectively. **DOI:** http://dx.doi.org/10.7554/eLife.21989.004

To establish CMI, several key issues as introduced earlier regarding its mechanisms of action and potential applications need to be clarified. We decided to employ homologous DCT_F_ from Ca_V_1.4 (α_1F_) with the strongest DCT tuning effects (stronger than DCT_D_) among the Ca_V_1 members (Figure 1—figure supplement 1), to construct the channel variant by fusing PCRD_F_-DCRD_F_ onto α_1DS_ right after IQ_V_ motif; meanwhile, since the potency of DCT effects is mainly determined by DCRD (Liu et al., 2010), PCRD from either Ca_V_1.3 or Ca_V_1.4 supposedly makes no or little difference and thus we did not distinguish these two PCRD isoforms in this work. Such channel variant was simply denoted as α_1DS_-PCRD-DCRD, which represents CMI intrinsic to the long variant of Ca_V_1.3 (α_1DL_) but with higher potency since strong DCRD_F_ replaces endogenous DCRD_D_. Compared with α_1DS_ control, *S_Ca_* and *J_Ca_* from α_1DS_-PCRD-DCRD were substantially attenuated, which provided ample dynamic ranges (highlighted by green areas) to explore CMI effects on CDI/VGA and its mechanisms (Figure 1B).

### Differential roles of DCRD and PCRD unveiled by low [apoCaM]

**Figure 2.**
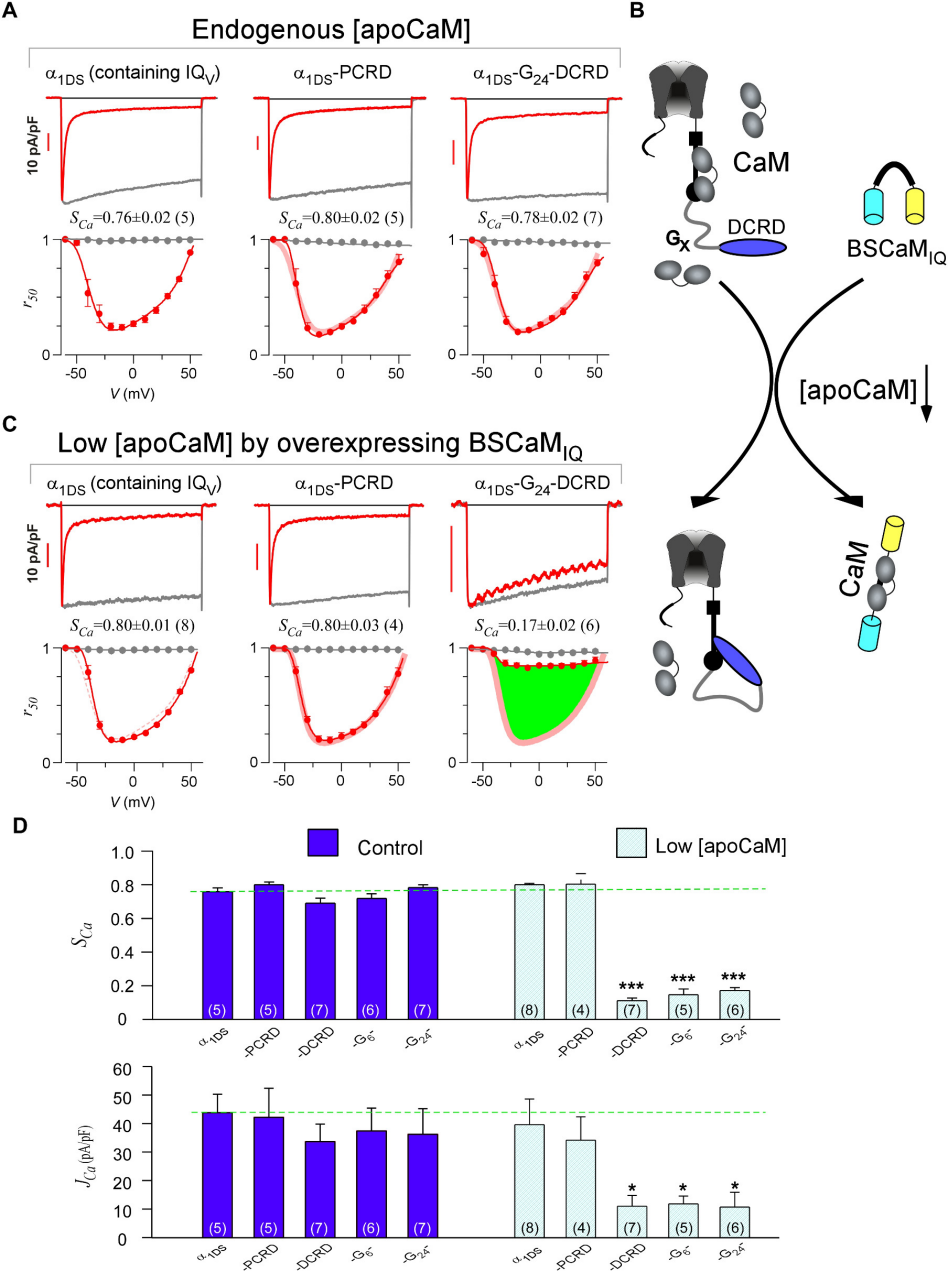
DCRD is indispensable and sufficient to induce CMI in low [apoCaM]. (**A**) All the channel variants including α_1DS_, α_1DS_-PCRD and α_1DS_-G_24_-DCRD exhibited strong CDI, indexed with *S_Ca_*. (**B**) Schematic illustration for the strategy to explore the minimum requirement of CMI. For α_1DS_-G_X_-DCRD containing glycine linkers (G_X_) of different length (0, 6 or 24), BSCaM_IQ_ that binds apoCaM was overexpressed to downregulate endogenous [apoCaM], in hope to promote the binding of DCRD with the channel (IQ_V_). (**C**) With low [apoCaM] by overexpressing BSCaM_IQ_, α_1DS_-G_24_-DCRD channels exhibited much attenuated CDI, evidenced by *I_Ca_* trace, *S_Ca_* value and *r_50_* profile (green area indicating the potency), in contrast to ultrastrong CDI of α_1DS_ or α_1DS_-PCRD, both lacking DCRD. (**D**) Statistical summary of CDI (*S_Ca_*) and VGA (*J_Ca_*) in endogenous (control) or low [apoCaM] for all channel variants of α_1DS_, α_1DS_-PCRD and α_1DS_-G_X_-DCRD, with additional information in Figure 2—figure supplement 1. Notably, for the three α_1DS_-G_X_-DCRD variants, both CDI and VGA were concurrently and significantly attenuated. Statistical significance was evaluated and indicated (p<0.001, ***; p<0.05, *). **DOI:** http://dx.doi.org/10.7554/eLife.21989.005

With such baseline CMI effects of PCRD-DCRD, we went further to examine whether both PCRD and DCRD are required, by fusing only PCRD or DCRD onto α_1DS_. Since DCRD might need some structural freedom as in DCT or PCRD-DCRD, a glycine linker of different length (G_X_) was inserted between DCRD and IQ_V_ to construct α_1DS_-G_X_-DCRD, mimicking the configurations that permit potent effects. From all these channel variants, we concluded no alteration in CDI in comparison with the α_1DS_ control, similarly from VGA results and analyses (Figure 2A and Figure 2—figure supplement 1A). In general, current densities should be more carefully scrutinized before any conclusive claim on VGA; and effects on *J_peak_* profiles and *J_Ca_* values normally need to be backed up by evaluations on concurrent CDI. For instance, current densities of α_1DS_-G_X_-DCRD (Figure 2—figure supplement 1A, the three columns on right) seemed different from the control, potentially interpreted as mild CMI; however, considering that the changes in CDI of these channels were rather small, the mild changes in VGA should be considered as variations instead of conclusive attenuations. Statistical test of significance (Student’s t-test) was performed subsequently to help identify significant CMI effects. Based on the fact that all the configurations under test failed to mediate *S_Ca_* and *J_Ca_* attenuations, all the three motifs of PCRD, DCRD and IQ_V_ might be required for effective CMI, consistent with the preliminary analyses on DCT subsegments from Ca_V_1.3 and Ca_V_1.4, in which the segment between PCRD and DCRD (‘B’ region) was dispensable but neither PCRD nor DCRD could be excluded (Liu et al., 2010).

**Figure 2—figure supplement 1.**
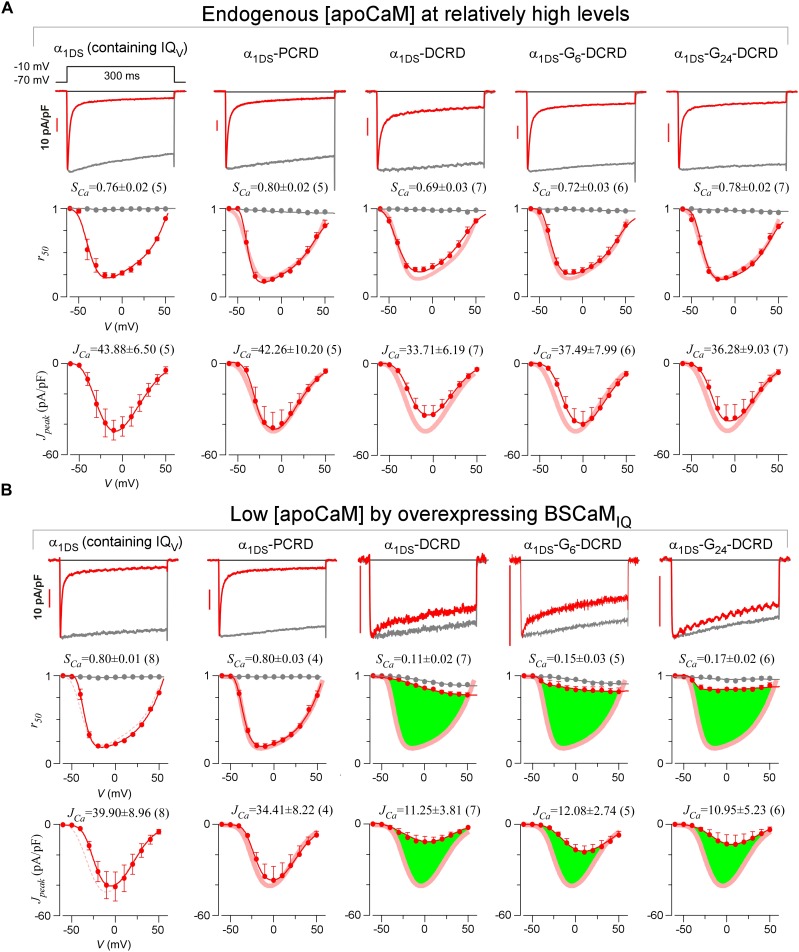
Full CDI and VGA profiles of different channel variants in endogenous and low [apoCaM]. (**A**) Under normal [apoCaM], channel variants of α_1DS_, α_1DS_-PCRD and α_1DS_-G_X_-DCRD (the number of glycine X equals 0, 6 and 24, respectively) exhibited no significant changes in exemplar *I_Ca_* traces (top row), CDI (middle row) and VGA (bottom row) profiles with respective indices of *S_Ca_* and *J_Ca_*. Small changes in VGA profiles for α_1DS_-G_X_-DCRD were within the experimental variations at least partially due to expression fluctuations in transient transfections; meanwhile, CDI, supposedly concurrently changing with VGA, was very close to the α_1DS_ control (thick semitransparent red lines). (**B**) When [apoCaM] was substantially lower down by overexpressing the apoCaM buffer of BSCaM_IQ_, α_1DS_-G_X_-DCRD exhibited potent attenuations on both CDI and VGA (green areas indicating the differences from the control group), but not for α_1DS_ or α_1DS_-PCRD containing no DCRD. Thick red lines (semitransparent) represent the *r_50_* and *J_peak_* profiles of the control group (α_1DS_ under low [apoCaM], left) for direct comparison. Dotted lines in the control group (left column) were replicated from α_1DS_ in normal [apoCaM] (**A**), essentially no difference. **DOI:** http://dx.doi.org/10.7554/eLife.21989.006

Notably, these channel variants expressed in HEK cells were quantified under normal concentrations of Ca^2+^-free CaM ([apoCaM]), in which the three-motif complex of IQ_V_/PCRD/DCRD as the core of CMI was perturbed by apoCaM that tends to pre-associate with IQ_V_ of the channel. To alleviate the interference with the potential cooperation in forming the threesome complex, we managed to substantially reduce [apoCaM] in the cells by overexpressing BSCaM_IQ_, a strong apoCaM buffer (Black et al., 2006), to help identify the minimum requirement for CMI (Figure 2B). In contrast to strong CDI in normal [apoCaM] evidenced from different channel variants (Figure 2A), with [apoCaM] being substantially reduced (but not lower than a realistic limit), surprisingly, α_1DS_-G_24_-DCRD and all the other DCRD-containing variants exhibited much attenuated CDI indicative of effective CMI (Figure 2C,D and Figure 2—figure supplement 1). Meanwhile, the stereotyped ultrastrong CDI normally observed from α_1DS_ or α_1DS_-PCRD was nearly unaltered by apoCaM buffers, in that under the residue [apoCaM] these channels were still able to be pre-bound with apoCaM considering the effective affinity between apoCaM and the channel (IQ_V_ or IQ_V_-PCRD) was reasonably high when there was no interference. Hence, mechanistically only DCRD and IQ_V_ are required by CMI so that PCRD becomes dispensable if [apoCaM] is low, unveiling the differential roles of PCRD and DCRD in the potential cooperation for CMI induction.

### Cooperative scheme of CMI consists of three major combinations

**Figure 3.**
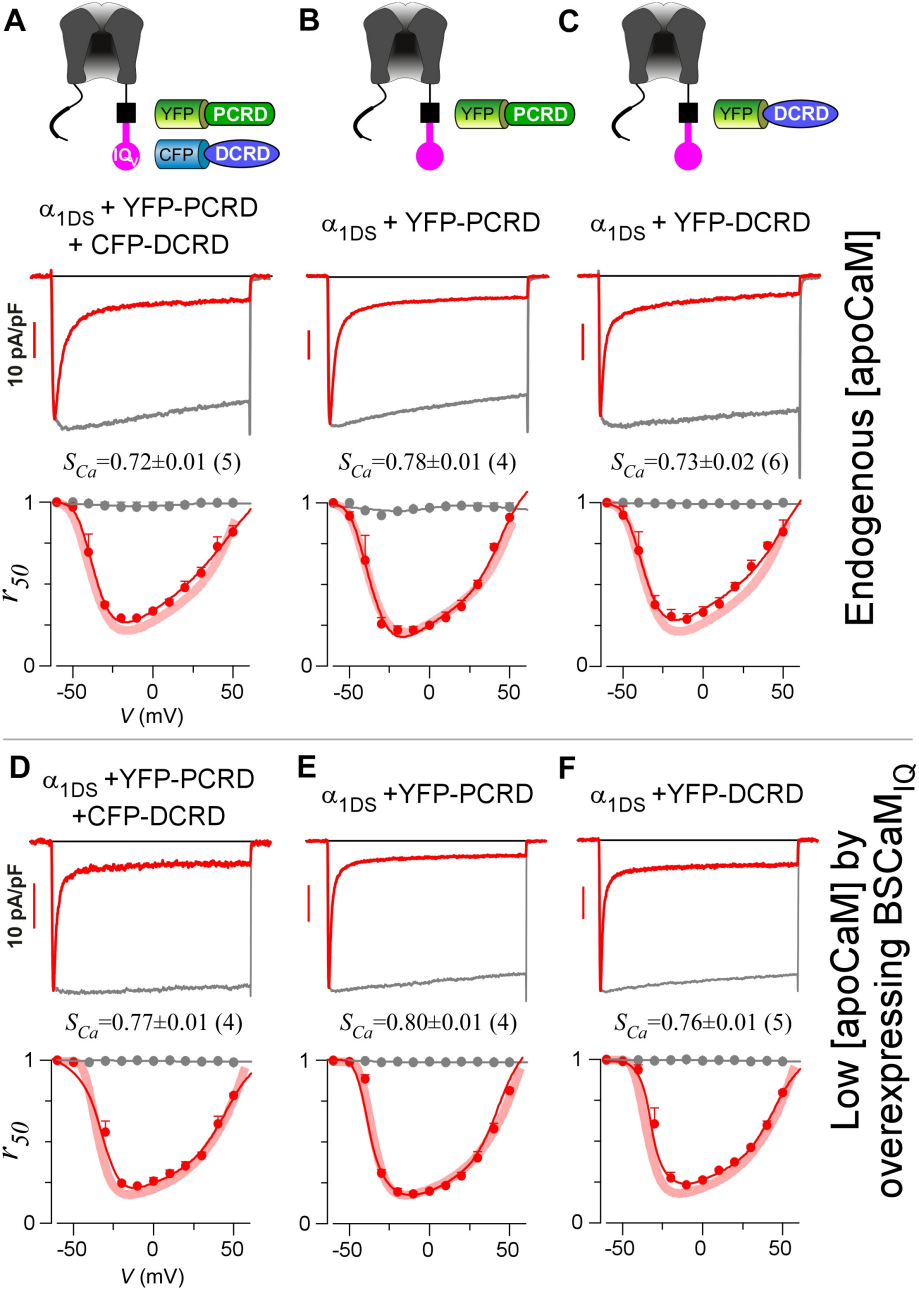
Individual DCRD peptides expressed separately from IQ_V_ are unable to induce CMI. (**A**) PCRD and DCRD tagged with fluorescent proteins were coexpressed with α_1DS_ as separate peptides. The presence of both YFP-PCRD and CFP-DCRD peptides in the same cell was confirmed under a fluorescence microscope (Figure 3—figure supplement 1). Under normal [apoCaM] in cells, no CMI effect was observed from *I_Ca_* trace exhibiting CDI similarly as α_1DS_ control, confirmed by comparable *S_Ca_* values and indistinguishable *r_50_* profiles. (**B** and **C**) Experiments and analyses were performed in normal [apoCaM] similarly as (**A**), except that only YFP-PCRD (**B**) or only YFP-DCRD (**C**) was expressed with α_1DS_ channels. Both resulted into strong CDI, with *S_Ca_* and *r_50_* indistinguishable from α_1DS_ control. (**D−F**) When free [apoCaM] was substantially reduced by overexpressing BSCaM_IQ_ (apoCaM buffers), the above three cases in (**A−C**) were re-examined. Exemplar *I_Ca_* traces exhibited strong CDI as quantified by *S_Ca_* values and *r_50_* profiles, similarly as the control group of α_1DS_ in low [apoCaM] (semitransparent line in red). **DOI:** http://dx.doi.org/10.7554/eLife.21989.007

The data thus far suggest that CMI mainly relies on DCRD/IQ_V_ binding to overcome free [apoCaM] of substantial level in normal cell conditions; and PCRD could facilitate CMI potentially by enhancing the binding of DCRD/IQ_V_ and thus the competition against apoCaM. However, the trio failed to attenuate CDI when they were separately expressed as three individual peptides (Figure 3A–C), even when PCRD, DCRD and IQ_V_ (contained in α_1DS_) were all well expressed in the same cell as confirmed with fluorescent tags (Figure 3—figure supplement 1). By examining VGA (*J_Ca_*) with aforementioned precautions, we also concluded that no CMI effect on α_1DS_ was detectable with all the three groups of peptides: PCRD and DCRD, PCRD only, and DCRD only. When [apoCaM] was reduced with chelator BSCaM_IQ_ as in Figure 2, CDI remained ultrastrong and indistinguishable from α_1DS_ control for all the three test groups (Figure 3D–F), and VGA analyses came to the same conclusions consistently (Figure 3—figure supplement 2D–F).

**Figure 3—figure supplement 1.**
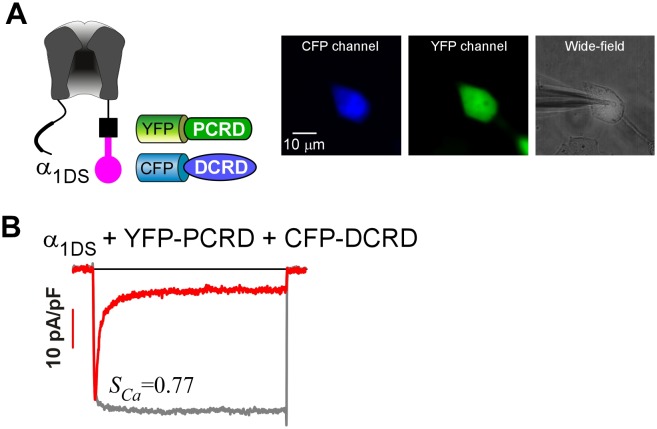
To confirm the presence of both PCRD and DCRD peptides in single cells. (**A**) YFP-PCRD and CFP-DCRD were coexpressed with α_1DS_ (left). For one single cell under patch recording (right image), the epi-fluorescence of high intensity via CFP (left image) and YFP (middle image) channels was observed to confirm that both peptides were well expressed. (**B**) The exemplar recording of *I_Ca_* from that particular cell in (**A**) exhibited strong CDI (*S_Ca_*), within the range of standard *S_Ca_* for α_1DS_ (0.76 ± 0.02, n = 5). **DOI:** http://dx.doi.org/10.7554/eLife.21989.008

**Figure 3—figure supplement 2.**
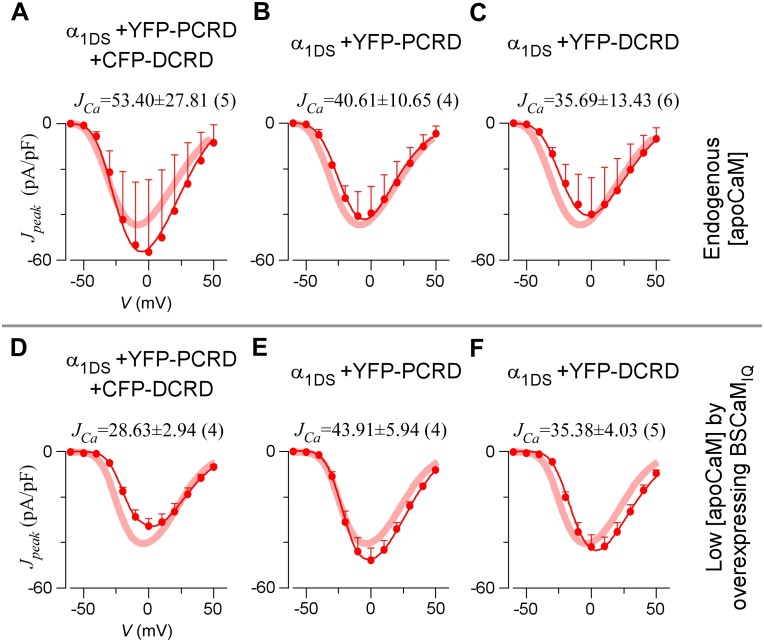
PCRD and DCRD as separate peptides are unable to inhibit VGA. (**A–C**) In normal [apoCaM], when both PCRD and DCRD were expressed but as separate peptides (**A**), the VGA profile of α_1DS_ (control, red thick semitransparent lines) was unaltered as indexed by *J_Ca_*. Similarly, no inhibition of VGA was observed when only PCRD (**B**) or only DCRD (**C**) was transfected. (**D−F**) When [apoCaM] was reduced by overexpressing BSCaM_IQ_, for all the three cases in (**A−C**), their VGA profiles were indistinguishable from the control group (red thick semitransparent lines), also confirmed by similar *J_Ca_* values across different groups. **DOI:** http://dx.doi.org/10.7554/eLife.21989.009

**Figure 4.**
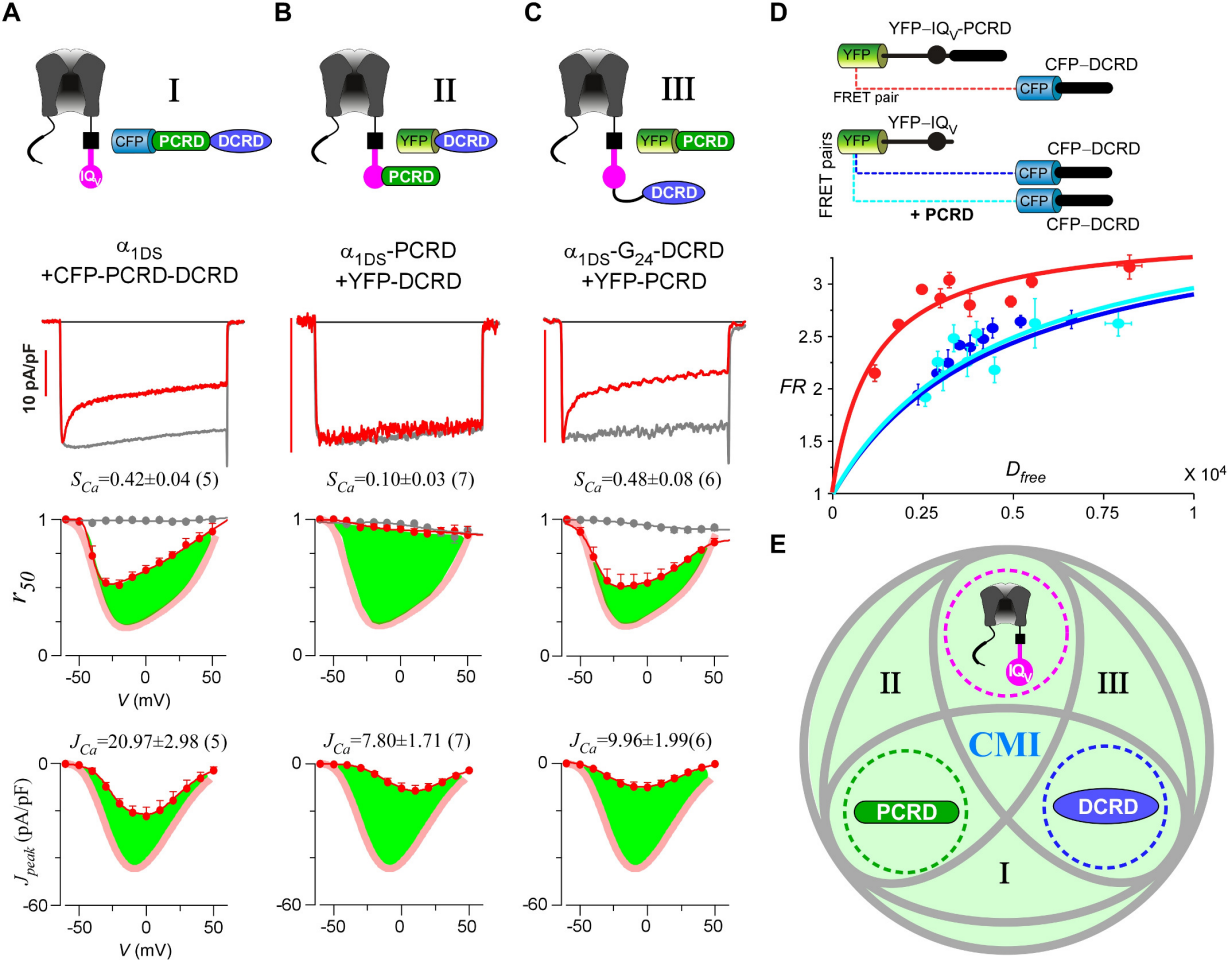
Cooperation by PCRD, DCRD and IQ_V_ to induce CMI. (**A**) Both CDI and VGA of α_1DS_ channels were attenuated by pre-linked PCRD-DCRD illustrated in the scheme (top), shown in the exemplar traces (middle) and voltage-dependent *r_50_* and *J_peak_* profiles (lower two panels). The potency of CMI was indexed by *S_Ca_* and *J_Ca_* values and also illustrated by the green areas. Profiles of α_1DS_ control were indicated by thick semitransparent lines in red (lower two panels). (**B**) Both CDI and VGA of α_1DS_-PCRD were strongly prohibited by DCRD. (**C**) Both CDI and VGA of α_1DS_-G_24_-DCRD were attenuated by PCRD. (**D**) FRET 2-hybrid assays demonstrated that pre-linked IQ_V_-PCRD motif (YFP tagged) exhibited strong binding with CFP-DCRD, with higher binding affinity (*K_d_* = 1135, units in donor-cube fluorescence intensity) than the binding affinity (*K_d_* = 4700) between CFP-DCRD and YFP-IQ_V_ itself (without PCRD being fused). Additional PCRD peptides did not make any appreciable change (*K_d_* = 4624) for the binding between CFP-DCRD and YFP-IQ_V_, unable to rescue the low affinity back to the high level that the constitutive PCRD fusion (YFP-IQ_V_-PCRD) could achieve. *FR_max_* values for all these binding curves were similar (3.50—3.87). Each data point represented the *FR* (y-axis, FRET ratio) and *D_free_* (x-axis, free donor concentration) values averaged over five adjacent individual cells sorted by *D_free_*. (**E**) Working model to illustrate the collaboration among three components of PCRD, DCRD and IQ_V_ (embedded within α_1DS_) for CMI induction. Grey circles represent the effective combinations, which require that at least two (out of three) components are closely engaged (*e.g.*, fusion) to form the trio complex incorporating the third (separate) component. The three key combinations are I (IQ_V_+PCRD-DCRD), II (IQ_V_-PCRD+DCRD) and III (IQ_V_-DCRD+PCRD), where ‘–’ denotes fusion or spatial closeness and ‘+’ indicates the separate peptide to be coexpressed. In addition, the positive control (IQ_V_-PCRD-DCRD) also represents the native long variant α_1DL_; and the three components (dotted circles in green, pink and blue) completely separate to each other (IQ_V_+PCRD+DCRD) produce no CMI effect, serving as one negative control. **DOI:** http://dx.doi.org/10.7554/eLife.21989.010

Up to here, we established two extreme cases for CMI, *i.e.*, either all the three motifs were fused together (strong CMI), or the three were totally isolated to each other (no CMI). To explore intermediate conditions between the two extremes, we physically linked every two modules out of the three to coexpress with the third one, yielding three possible combinations in total. And they were, PCRD-DCRD peptides to coexpress with IQ_V_ (contained in α_1DS_) (Figure 4A, combination I), channels containing IQ_V_-PCRD to coexpress with DCRD (Figure 4B, combination II), and channels containing linked IQ_V_ and DCRD to coexpress with PCRD (Figure 4C, combination III). Interestingly, all the three combinations exhibited CMI, strongly attenuating both CDI and VGA as illustrated by green areas indicating the altered *r_50_* and *J_peak_* profiles compared to α_1DS_ control (Figure 4A—C). Combination I also provided a great chance to revisit the assumption we made earlier regarding interchangeable PCRD_D_ and PCRD_F_ from Ca_V_1.3 and Ca_V_1.4, respectively. Instead of dealing with two different channel variants, here the same α_1DS_ served as the control to reliably evaluate PCRD_D_-DCRD *vs* PCRD_F_-DCRD peptides. And our assumption was validated by the negligible differences in VGA and CDI between the two PCRD-DCRD peptides (Figure 4—figure supplement 1). In previous reports, combinations of I and II were already suggested when studying endogenous DCT in Ca_V_1.3 (α_1DL_) and Ca_V_1.4 (Singh et al., 2008, 2006). In addition, PCRD (combination III) was able to induce pronounced CMI effects as we first observed in this work, which also completed all the three possible combinations of peptide-mediated CMI.

**Figure 4—figure supplement 1.**
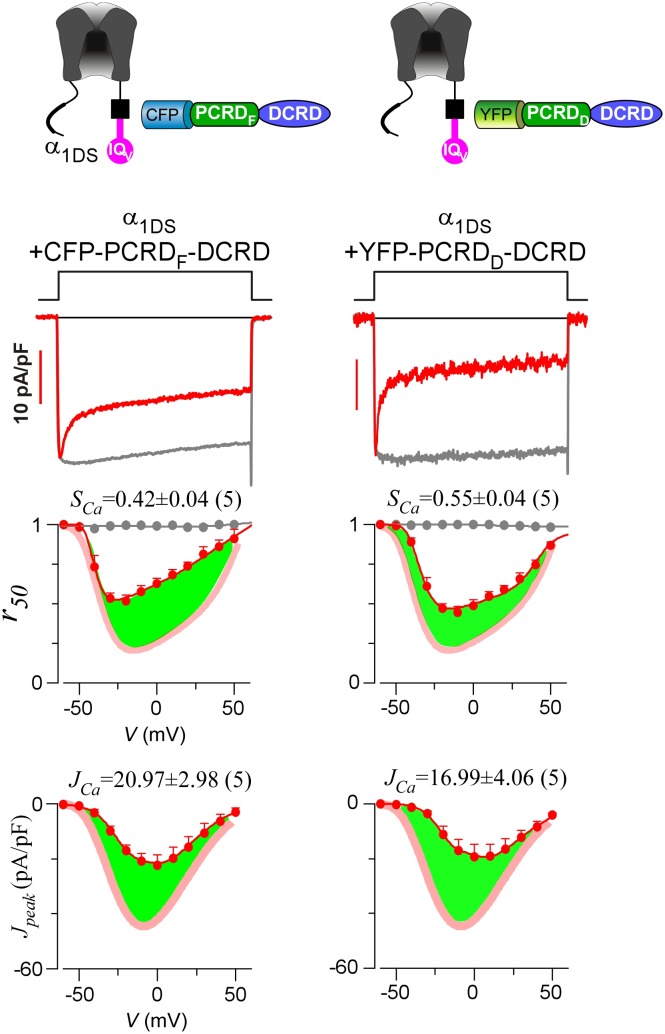
Functional similarities between PCRD_F_ and PCRD_D_. The pronounced CDI (*S_Ca_*) and strong VGA (*J_Ca_*) of α_1DS_ channels (indicated by red thick semitransparent lines in *r_50_* and *J_peak_* plots) were similarly attenuated by PCRD-DCRD peptides made from either PCRD_F_ from Ca_V_1.4 (left column) or PCRD_D_ from Ca_V_1.3 (right column). Representative *I_Ca_* traces (top), CDI and VGA profiles (panels in the two bottom rows) across the full voltage range were shown for the two types of peptides, with no significant difference. Green areas represent the potency of attenuation, supporting that PCRD_F_ and PCRD_D_ were interchangeable for CMI in this study as we assumed. **DOI:** http://dx.doi.org/10.7554/eLife.21989.011

**Figure 4—figure supplement 2.**
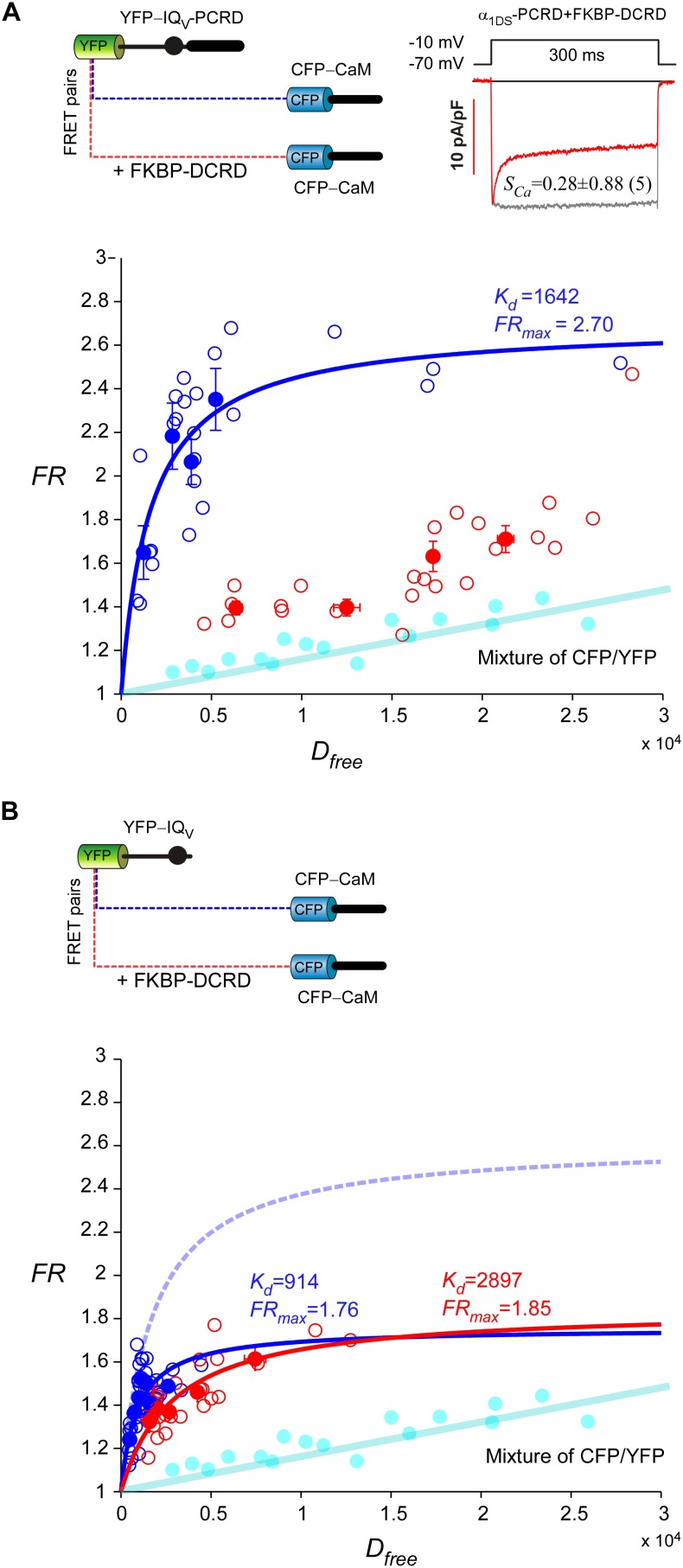
Cooperative perturbation of apoCaM/IQ_V_ binding by DCRD and PCRD. (**A**) By 2-hybrid FRET with the CFP- or YFP-labeled constructs illustrated on top, IQ_V_-PCRD (YFP-tagged) and CaM (CFP-tagged, apo-state) exhibited strong binding (blue), quantified by the maximum FRET ratio (*FR_max_*) and effective equilibrium dissociation constant (*K_d_*) from iterative curve fitting. The values of *FR_max_* and *K_d_* were indicated right above the fitted curves (in the same color) when applicable. Unfilled-dots and filled-dots represent individual cells and averaged results (over five cells) respectively. The binding between apoCaM and IQ_V_-PCRD were severely perturbed by FKBP-DCRD (with FKBP tag adopted from subsequent rapamycin-inducible CMI, but without fluorescent tags of CFP/YFP), resulted into the data points (red) approaching the baseline of CFP/YFP mixture (cyan), indicative of very weak *K_d_*. The perturbation was presumably by way of the close cooperation between FKBP-DCRD and IQ_V_-PCRD to compete against apoCaM. The FRET binding analyses were consistent with the patch-clamp recordings confirming that CDI (*S_Ca_*) of α_1DS_-PCRD was attenuated in the presence of FKBP-DCRD (inset). (**B**) In the absence of PCRD, the perturbation by FKBP-DCRD was much less effective. Without the presence of PCRD, CFP-CaM and YFP-IQ_V_ (blue) were still able to bind. However, in contrast to the strong perturbation seen in (**A**), in the absence of PCRD, inclusion of DCRD peptides made no or very little difference in *K_d_* and *FR_max_*values for CaM and IQ_V_. The binding curve (dotted line in light blue) was replicated from CFP-CaM and YFP-IQ_V_-PCRD (**A**) for comparison. **DOI:** http://dx.doi.org/10.7554/eLife.21989.012

Live-cell FRET two-hybrid assays demonstrated that DCRD bound IQ_V_-PCRD with higher affinity (*K_d_* = 1135, units in donor-cube fluorescence intensity) than that between DCRD and IQ_V_ (*K_d_* = 4700), regardless of whether PCRD as the standalone peptide was also present (*K_d_* = 4624) (Figure 4D). Owing to the high affinity between DCRD and IQ_V_-PCRD, DCRD overexpression nearly abolished the binding between apoCaM and IQ_V_-PCRD (*i.e.*, apoCaM/channel pre-association) as demonstrated in the plot of *FR-D_free_* (FRET ratio *vs* free donor concentrations) (Figure 4—figure supplement 1A); meanwhile, without the presence of PCRD, apoCaM was still able to bind IQ_V_, but the DCRD perturbation was much less effective (Figure 4—figure supplement 1B). The electrostatic interaction between PCRD and DCRD hinted by Ca_V_1.2 (Hulme et al., 2006) seemed generalizable but was not detected with the FRET pairs based on Ca_V_1.4 DCT (Liu et al., 2010). Both this putative PCRD/DCRD interaction and the binding between IQ_V_ and DCRD should be weaker and secondary compared to the strong binding between IQ_V_-PCRD and DCRD (Figure 4D). This explained why the potential contributions of these secondary interactions may or may not be evidenced, depending on whether the trio complex would come into being in particular settings. Our FRET binding analyses are consistent with the functional data shown earlier, thus providing additional information for the collaborations among CMI motifs. For instance, coexpression of all the three but separate components would not produce CMI (Figure 3A). Also, DCRD strongly attenuated α_1DS_-PCRD but not α_1DS_ control (Figure 4B *vs* Figure 3C).

Taken together, a cooperative scheme of CMI under physiological [apoCaM] in the cell was unveiled by functional and binding analyses (Figure 4E), which depicted that if any two components have enough spatial closeness or intimacy, *e.g.*, by prearranged linkage, it would be sufficient for the third component to form the ‘trio’ complex and thus induce effective CMI (combinations of I, II and III, highlighted in grey ellipses). Also, the fusion of all the three components together mimics the intrinsic CMI in α_1DL_ (positive control, Figure 1B); and expression of three components separate to each other represents the negative control with no CMI (Figure 3A).

### Acute CMI based on rapamycin-inducible heterodimerization and cooperation

**Figure 5.**
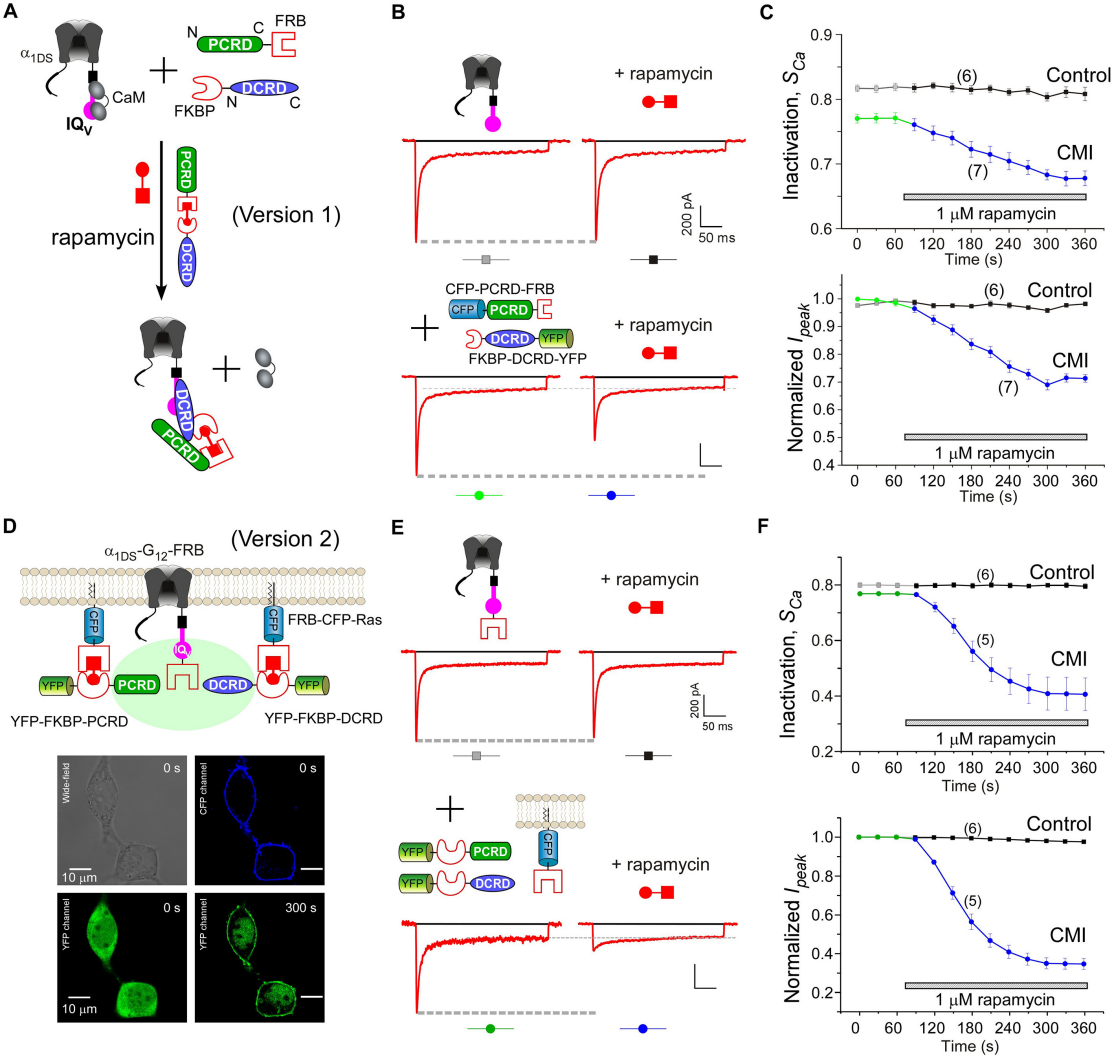
Design schemes and experimental implementations of rapamycin-inducible CMI. (**A**) Design principles for chemical-inducible CMI. Rapamycin simultaneously binds one FKBP and one FRB to combine any two FRB/FKBP-tagged components (PCRD and DCRD, in this version 1) selected from the three components of PCRD, DCRD and IQ_V_. Thus, the three components would form the combinations (combination I, in the version 1) to satisfy the requirement of cooperative CMI (Figure 4E). (**B**) One representative implementation of chemical-inducible CMI. According to the design of version one in (**A**), PCRD-FRB and FKBP-DCRD were constructed and coexpressed with α_1DS_. Exemplars of Ca^2+^ current traces demonstrated that acute effects were induced by applying 1 μM rapamycin to the multi-component system of version 1 (lower panel), in contrast to α_1DS_ control with no appreciable changes in *I_Ca_* (upper panel). (**C**) Statistical summary of rapamycin-induced CMI (version 1). Averaged values over multiple cells (number indicated in parentheses) for the indices *S_Ca_* (upper) or normalized *I_peak_* (lower) demonstrated time-dependent attenuations on both CDI and VGA upon rapamycin application as compared to the control group. (**D**) Rapamycin perfusion induced rapid translocation of YFP-FKBP-DCRD or YFP-FKBP-PCRD (version 2) onto the membrane by linking with FRB-CFP-Ras within 5 min, shown by confocal images via wide-field, CFP, and YFP channels. The local concentrations of YFP-FKBP-DCRD and YFP-FKBP-PCRD were substantially enhanced as suggested by the condensed YFP fluorescence at the membrane (outlined by CFP fluorescence). (**E** and **F**) According to rapamycin-inducible CMI of version 2, recombinant channels of α_1DS_-G_12_-FRB were coexpressed with cytosolic YFP-FKBP-PCRD and YFP-FKBP-DCRD. To enhance local concentrations of FKBP-tagged peptides near the channels, membrane-localized FRB-CFP-Ras was also overexpressed. Exemplars of Ca^2+^ current traces (**E**) exhibited strong attenuation (lower), in contrast to stable *I_Ca_* from α_1DS_-G_12_-FRB alone (upper). In contrast to the control group, temporal profiles of rapamycin-inducible CMI (version 2) indicated strong attenuations on CDI (*S_Ca_*, upper) and VGA (normalized *I_peak_*, lower). Based on totally five trials (cells), *S_Ca_* changed from 0.77 ± 0.00 to 0.41 ± 0.06 and *I_peak_* was reduced to 35% ± 3% of the basal levels (**F**). **DOI:** http://dx.doi.org/10.7554/eLife.21989.013

Based on such cooperation-dependent CMI (Figure 4E) and rapamycin-mediated heterodimerization (Yang et al., 2013, 2007), we devised the strategy to implement drug-inducible CMI, illustrated in the design of version 1 (Figure 5A). FKBP (rapamycin-binding protein) and FRB fragment (FKBP and rapamycin binding domain of the kinase mTOR) as the tags were fused onto DCRD and PCRD, respectively. A small immunosuppressant molecule rapamycin that binds both FRB and FKBP was applied to link one FKBP-DCRD together with one PCRD-FRB, aiming to bind to the IQ_V_ domain of α_1DS_ to compete off apoCaM and thus induce CMI according to the scheme of combination I (Figure 4E). This design, if successful, should be directly applicable to native Ca_V_1.3 since no modification is needed at the channel side. Attracted by such potentials, we implemented and validated this rapamycin-inducible peptide-mediated CMI in HEK cells with α_1DS_. In contrast to the stable time-course profiles of the control group (Figure 5B), CDI (*S_Ca_*) and VGA (*I_peak_*) were both rapidly attenuated upon applying 1 μM rapamycin. Within tens of seconds, dimerization between FKBP-DCRD with PCRD-FRB started to attenuate *I_Ca_* as evidenced by time-dependent decays (Figure 5C).

**Figure 5—figure supplement 1.**
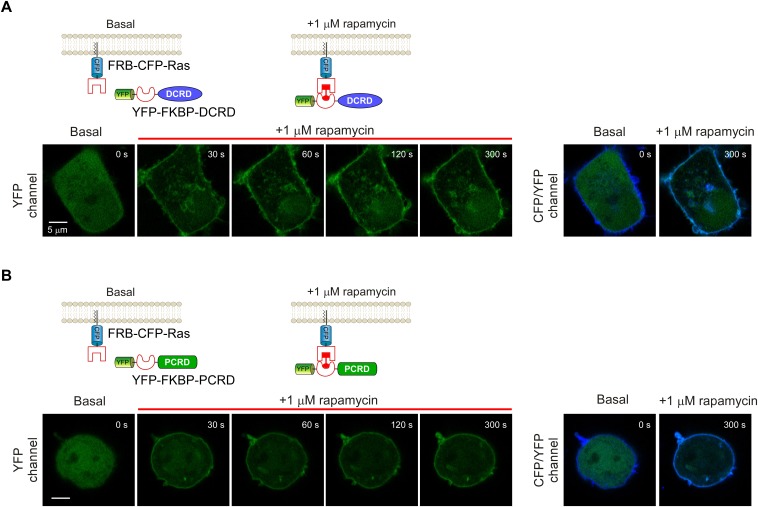
Temporal profiles of fluorescent images for rapamycin-induced membrane-targeting. (**A** and **B**) Top rows: cartoon depicting the design of the rapamycin-induced translocation of DCRD (**A**) or PCRD (**B**) (Version 2 of chemical-inducible CMI). FRB-CFP-Ras was constitutively anchored to the plasma membrane by incorporating membrane-targeting segment from Ras protein. Lower rows: confocal images acquired via YFP channel showing rapid translocation of YFP-FKBP-DCRD (**A**) or YFP-FKBP-PCRD (**B**) to plasma membrane upon 1 μM rapamycin perfusion (left). At the time of ~120 s or later, YFP fluorescence was substantially condensed to overlap with the stable CFP fluorescence (FRB-CFP-Ras) on the membrane (right). **DOI:** http://dx.doi.org/10.7554/eLife.21989.014

**Figure 5—figure supplement 2.**
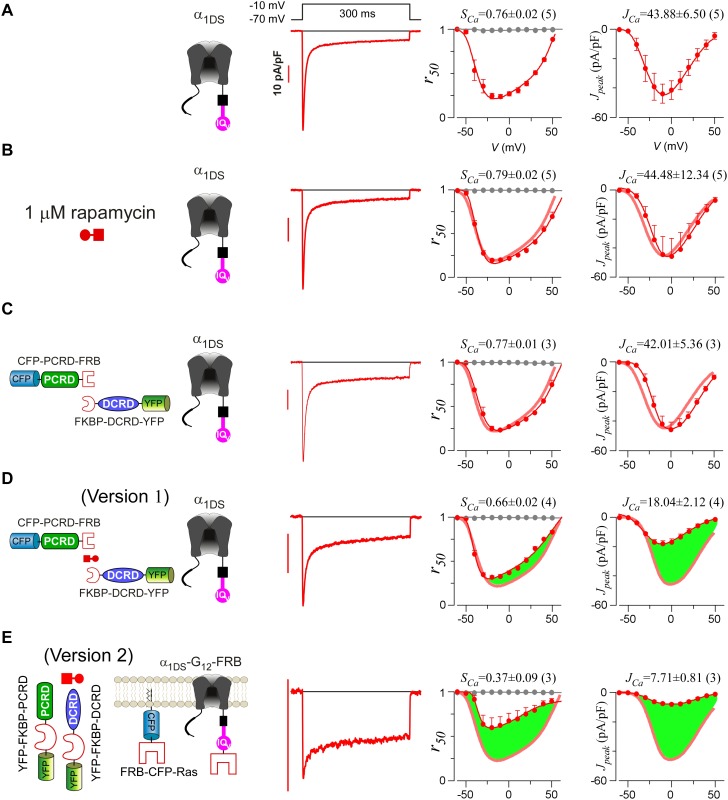
Detailed characterizations for rapamycin-induced CMI. (**A** and **B**) Voltage-dependent profiles of CDI and VGA exhibited no difference when α_1DS_ channels were with (**B**) or without (**A**) rapamycin. Thick lines (red semitransparent) represent the CDI and VGA profiles from the α_1DS_ control group. (**C**) Coexpression of CFP-PCRD-FRB and FKBP-DCRD-YFP with α_1DS_ representing the control conditions before rapamycin did not cause any appreciable change in CDI and VGA, confirmed by pronounced *S_Ca_* and *J_Ca_* comparable to the values from α_1DS_ control. (**D**) When rapamycin was applied to α_1DS_ coexpressed with CFP-PCRD-FRB and FKBP-DCRD-YFP, *S_Ca_* and *J_Ca_* averages over different cells were attenuated with moderate potency (illustrated by green areas) presumably by rapamycin-induced linkage between PCRD and DCRD (version 1 of inducible CMI) to form the cooperation for effective CMI. (**E**) In rapamycin, with enhanced local concentrations of YFP-FKBP-PCRD and YFP-FKBP-DCRD (version 2 of rapamycin-induced CMI), the potency was much improved, demonstrated by more pronounced attenuations concurrently on both CDI and VGA (larger green areas in both *r_50_* and *J_peak_* profiles). **DOI:** http://dx.doi.org/10.7554/eLife.21989.015

Encouraged by the first prototype of inducible CMI, we then proceeded to enhance the potency from moderate (as in version 1) to ultrastrong attenuation similarly as in Figure 1B. We reasoned that the underperformance here should be mainly due to the low effective concentration of the linked peptides if closely comparing constitutive (Figure 4A) and the rapamycin-inducible CMI (Figure 5C). The upgrade version (version 2) was designed to enhance concentration (local to channels) and thus the potency of CMI peptides. Membrane-targeting motif Ras (Yang et al., 2007) was used to construct FRB-CFP-Ras, which would bind DCRD/PCRD tagged with FKBP through rapamycin-inducible heterodimerization. As demonstrated by confocal fluorescence imaging, in about two minutes YFP-FKBP-DCRD and YFP-FKBP-DCRD translocated to the membrane upon rapamycin application (Figure 5D, Figure 5—figure supplement 1). Meanwhile, FRB was also fused onto the channel to construct α_1DS_-G_12_-FRB for inducible dimerization with FKBP-PCRD or FKBP-DCRD, to facilitate peptide-mediated CMI according to the schemes of combination II and III (Figure 4E, Figure 5D). More potently than the earlier design but in a similar time course (version 1, Figure 5B,C), rapamycin induced strong CMI effects on *I_Ca_* within 4–5 min (version 2, Figure 5E,F). Both CDI and VGA were substantially attenuated: *S_Ca_* (0.41 ± 0.06, n = 5) and *I_peak_* (35% ± 3%, n = 5). Both indices reached the plateau within 4–5 min, which was about the speed of full solution exchange in our recording system, indicating that rapamycin perfusion was the major time-limiting factor. Thus, the acute nature of CMI was strongly supported by the temporal profiles of inducible CMI effects on CDI/VGA (both version 1 and version 2).

To further consolidate the results, direct drug-channel effects were examined for rapamycin as the negative controls; and full CDI and VGA profiles were characterized across the whole voltage range before and after applying rapamycin (Figure 5—figure supplement 2).

### CMI is a unique type of inhibition but sharing similarities with CDI

**Figure 6.**
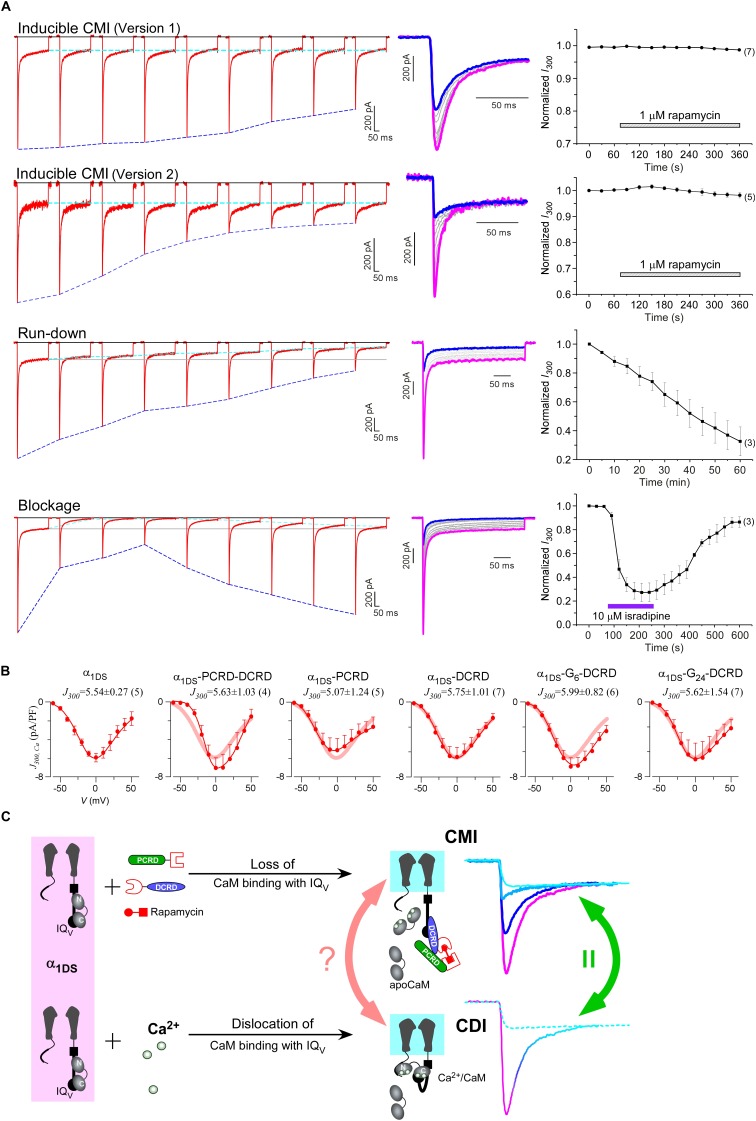
Mechanistic insights enlightened by unique features of CMI. (**A**) Temporal profiles between CMI and conventional inhibitions were compared. For rapamycin-inducible CMI (two versions in the top two rows of panels), representative Ca^2+^ current traces in rapamycin were selected from sequential time-points (left column) and superimposed together (middle column) for comparison. The time sequence was indicated by color: the first in pink, intermediate in grey, and the last in blue. *I_peak_* exhibited the trend of inhibition but not for *I_300_* (*I_Ca_* at 300 ms) (right column). In contrast, for run-down process and isradipine blockage (bottom two rows), both *I_peak_* and *I_300_* exhibited the declining trends indicating substantial inhibitions. (**B**) Ca^2+^ current density at 300 ms (*J_300,Ca_*) remained at the same level (indicated by the *J_300_* values at −10 mV), for various channel variants under test, regardless of whether CMI was in effect. Thick lines (semitransparent in red) represent the *J_300,Ca_* profile of α_1DS_ control. (**C**) Functional and the structural insights into the two modes of inhibition. DCT/apoCaM-dependent CMI and Ca^2+^/CaM-mediated CDI result in indistinguishable gating (green arrows) appearing due to similar causes, *i.e.*, either total or partial loss of the apo-state CaM/IQ_V_ complex. That says, in CMI, apoCaM pre-association is totally lost; and in CDI, apoCaM is calcified and dislocated from the pre-association sites. Although the triggers are different for CMI *vs* CDI, *i.e.*, DCT competing (off apoCaM) *vs* Ca^2+^ binding (onto apoCaM), the similarities between the two different inhibitory regulations of CMI and CDI invite the hypothesis that the core gating machinery (cyan squares) upon depolarization might step into the same scenario/mode including structural details (red arrows). A series of current traces (on the right) indicate CMI with different potency (enhanced from pink to cyan, upper), in comparison with the trace at different stages of CDI (developed from pink to cyan, lower) superimposed with the trace from ultrastrong CMI (dotted trace in cyan). These analyses point to one important notion that the lower limit of CMI is determined by end-stage CDI (the traces or the phases in cyan), and thus the dynamic changes for CMI effects on α_1DS_ (indexed with *I_peak_* and *S_Ca_*) are preset, *i.e.*, from the pink (no CMI and ultrastrong CDI) to the cyan (ultrastrong CMI and no CDI). **DOI:** http://dx.doi.org/10.7554/eLife.21989.016

One interesting feature we discovered from rapamycin-inducible CMI was that the current amplitude at 300 ms (*I_300_*) stayed at the same levels during the whole time course (Figure 6A, top two rows), in contrast to rapidly decayed *I_peak_* as outlined by blue dotted lines. In this context, CMI was quite unique compared to other conventional inhibitions. In the run-down process (Kepplinger and Romanin, 2005) or the blockage by isradipine (Anekonda and Quinn, 2011), the number of functional channels was reduced, which resulted into attenuations on *I_Ca_* during the whole depolarization step including both *I_peak_* and *I_300_* (Figure 6A, lower two rows). In contrast, *S_Ca_* remained constant for α_1DS_ undergoing run-down or blockage since intrinsic gating properties (*e.g.*, CDI) of each channel were not altered, whereas *S_Ca_* was attenuated in CMI (Figure 6—figure supplement 1A).

**Figure 6—figure supplement 1.**
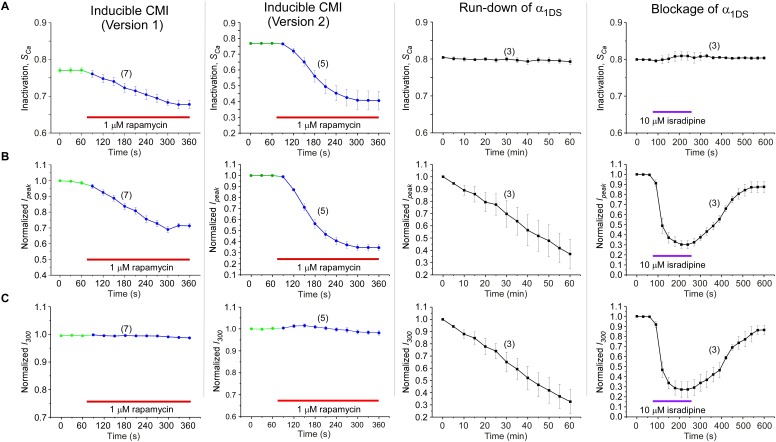
Detailed comparisons among CMI, run-down and blockage. CMI was compared with conventional inhibitions/inhibitors including channel blockage (by isradipine) and run-down process (reduction of channel numbers), indexed with *S_Ca_*, *I_peak_* and *I_300_*. Both rapamycin-induced CMI and run-down/blockage exhibited time-dependent decreases in *I_peak_*, as expected from effective *I_Ca_* inhibitions (**B**). In contrast, CDI (*S_Ca_*) did not change in run-down/blockage; whereas for CMI *S_Ca_* exhibited rapamycin-dependent attenuation, similar to *I_peak_* (**A**). Moreover, the plateau of *I_Ca_* (*I_300_*) remained constant in spite of *I_peak_* attenuation in CMI, whereas *I_300_* changed (decreased or recovered) in a similar time-course to that of *I_peak_* during run-down and blockage (**C**). Since *S_Ca_* is in proportion to *I_peak_*/*I_300_*, the two major types of inhibitions (CMI *vs* conventional inhibitions) were distinct within this context: for CMI, both *I_peak_* and *S_Ca_* changed but with *I_300_* being kept constant; in contrast, for conventional inhibitions, both *I_peak_* and *I_300_* changed, but *S_Ca_* remained unaltered. **DOI:** http://dx.doi.org/10.7554/eLife.21989.017

**Figure 6—figure supplement 2.**
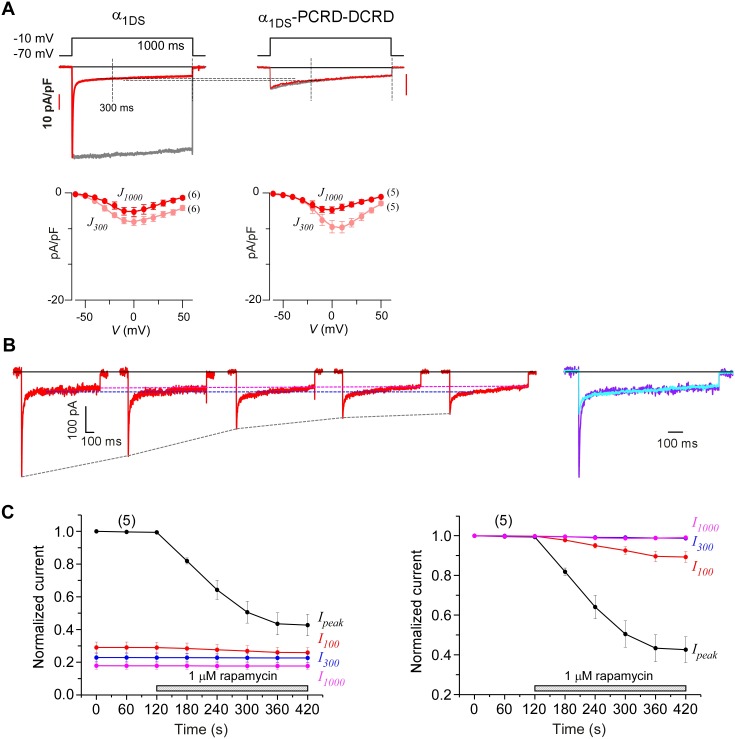
Indices at different time points of *I_Ca_* to quantify CMI and end-stage CDI. (**A**) Analyses at both 300 ms and 1000 ms were able to reliably unveil that channels subject to CMI were essentially tuned to approach *I_Ca_* levels in end-stage CDI. Upper panel, we set *I_1000_* (*I_Ca_* at 1000 ms) of both exemplar traces to the same level for α_1DS_ and α_1DS_-PCRD-DCRD (in principle, they should be the same); and the levels of *I_Ca_* at 300 ms (*I_300_*) turned out to be nearly the same, similarly as the analyses with *I_1000_*. At 300 ms, the kinetic difference between *I_Ba_* (normalized to the peak of *I_Ca_*) and *I_Ca_* was very little, indicative of end-stage CDI (upper right). Full profiles of Ca^2+^ current density (indexed with *J_300_* or *J_1000_*) were indistinguishable for both groups of α_1DS_ and α_1DS_-PCRD-DCRD, further supporting that both are qualified as reliable indices for CMI analysis. (**B**) For rapamycin-inducible CMI (version 2) by supplying PCRD, DCRD and α_1DS_ with membrane-targeting and rapamycin-inducible mechanisms, representative *I_Ca_* traces were selected from sequential time-points (left), and the first (purple) and the last (cyan) traces were superimposed together for comparison (right). *I_peak_* exhibited a trend of time-dependent decrease; meanwhile, both *I_300_* and *I_1000_* remained rather constant (dotted outlines in black, blue and pink respectively). (**C**) Statistical summary of *I_Ca_* amplitudes for rapamycin-induced CMI (version 2). *I_Ca_* values were measured at different time points, *i.e.*, *I_peak_*, *I_100_*, *I_300_* and *I_1000_*, each of which was either rescaled to *I_peak_* (left) or to its own (right). *I_peak_* exhibited robust inhibition due to CMI, and a trend of moderate decline (right) was noticeable from *I_100_* (*I_Ca_* at 100 ms); whereas *I_300_* and *I_1000_* clearly remained constant throughout the full time-course, thus reliably representing the end stage or steady state of CDI. In line with our previous analyses, the current level of *I_300_* or *I_1000_* determined the lower limit of *I_Ca_* (measured by *I_peak_*) subject to CMI of the maximum potency. **DOI:** http://dx.doi.org/10.7554/eLife.21989.018

**Figure 6—figure supplement 3.**
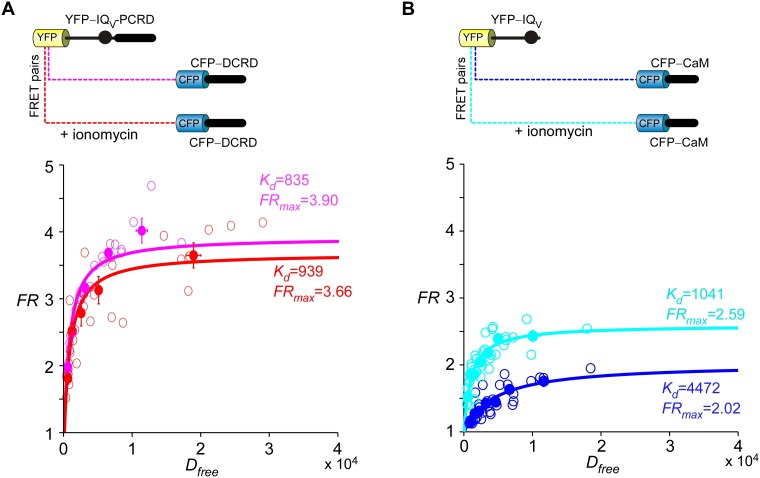
The high-affinity binding between DCRD and the channel was not perturbed by Ca^2+^/CaM. (**A**) Upon applying ionomycin, an ionophore massively raising intracellular Ca^2+^ levels, endogenous CaM should switch from Ca^2+^-free state (apoCaM) to Ca^2+^-bound state (Ca^2+^/CaM). However, similar *K_d_* values were obtained by fitting the FRET binding curves before and after ionomycin application (pink, before ionomycin; red, in ionomycin), indicating that Ca^2+^/CaM was unable to perturb the strong binding between DCRD and IQ_V_-PCRD. In the *FR-D_free_* plots, unfilled-dots and filled-dots represent individual cells and averaged results (over five cells) respectively. (**B**) Following ionomycin administration, the indices (*K_d_* and *FR_max_*) of the binding between CaM and IQ_V_ were clearly strengthened consistent with the IQ_V_/CaM interactions previously established. **DOI:** http://dx.doi.org/10.7554/eLife.21989.019

Would such constant *I_300_* be just a coincidence? By revisiting different channel variants tested earlier, we concluded that *I_300_* indeed remained at similar amplitudes for them all (Figure 6B), whereas their *I_peak_* and *S_Ca_* exhibited broad dynamic ranges. To confirm, depolarization pulses of longer duration (1000 ms) were also utilized to conduct constitutive and rapamycin-inducible CMI (Figure 6—figure supplement 2). In both modalities of CMI, the behavior of *I_300_* was similar to *I_1000_* and both indices remained constant. In addition, CDI already approached its steady-state at 300 ms (end-stage CDI), and the slight difference between *I_300_* and *I_1000_* was largely due to VDI and negligible in this study. Throughout this work, *I_300_* was employed as the major index to for both CMI and CDI analyses.

The constant *I_300_* (or *J_300_*) essentially set the lower limit for CMI. That said, while CMI potency was getting higher, *J_peak_* was tuned down to more closely approach *J_300_* (*e.g.*, Figure 6B *vs* Figure 1B). More clear evidence came from rapamycin-inducible CMI: the attenuated *I_peak_* and constant *I_300_* altogether account for *S_Ca_* (CDI) attenuation; and for ultrastrong CMI, *S_Ca_* would be nearly abolished (0), indicating that *I_peak_* would reach approximately the level of its lower limit (*I_300_*).

These lines of evidence also led us to conclude that a reduction of Ca^2+^ influx is guaranteed for CMI, despite concurrent VGA/CDI (activation/inactivation) attenuations. Such concurrency apparently would cause contradictory effects on Ca^2+^ influx: inhibition of CDI (ICDI) would tend to enhance Ca^2+^ influx, in opposition to attenuation on VGA (less Ca^2+^ influx). Even though, we just clarified that CMI attenuation on CDI is actually realized by reducing *I_peak_* while maintaining the end-stage *I_Ca_* (indexed with *I_300_* or *I_1000_*), which ensures the overall Ca^2+^ influx is reduced. Thus, the major uncertainty was relieved for CMI to emerge as a one new modality of Ca^2+^ channel inhibition.

These observations are consistent with the speculation that CDI might be the reversed process to the apoCaM promotion of open probability (Adams et al., 2014). High similarities are shared by DCT/apoCaM-dependent CMI and Ca^2+^/CaM-mediated CDI: functionally CMI (ultrastrong) and CDI (end-stage) could result into similar gating; and mechanistically they appear to be triggered by similar events: the pre-association between apoCaM and IQ_V_ is either totally abolished (CMI) or drastically altered (CDI) (Figure 6C). These facts suggest that potentially the same set of ‘core machinery’ (cyan square) mediates both CMI and CDI with very similar structures and structural changes. To exclude potential complications from ambient Ca^2+^/CaM, we performed FRET experiments to ensure that the interactions within the trio complex of IQ_V_/PCRD/DCRD were largely unaffected by Ca^2+^/CaM produced from ionomycin-introduced Ca^2+^ and endogenous CaM, although Ca^2+^/CaM indeed exhibited higher binding affinity to IQ_V_ than apoCaM (Figure 6—figure supplement 3).

### CMI inhibits Ca_V_1.3-mediated oscillation and pacemaking in SNc neurons

**Figure 7.**
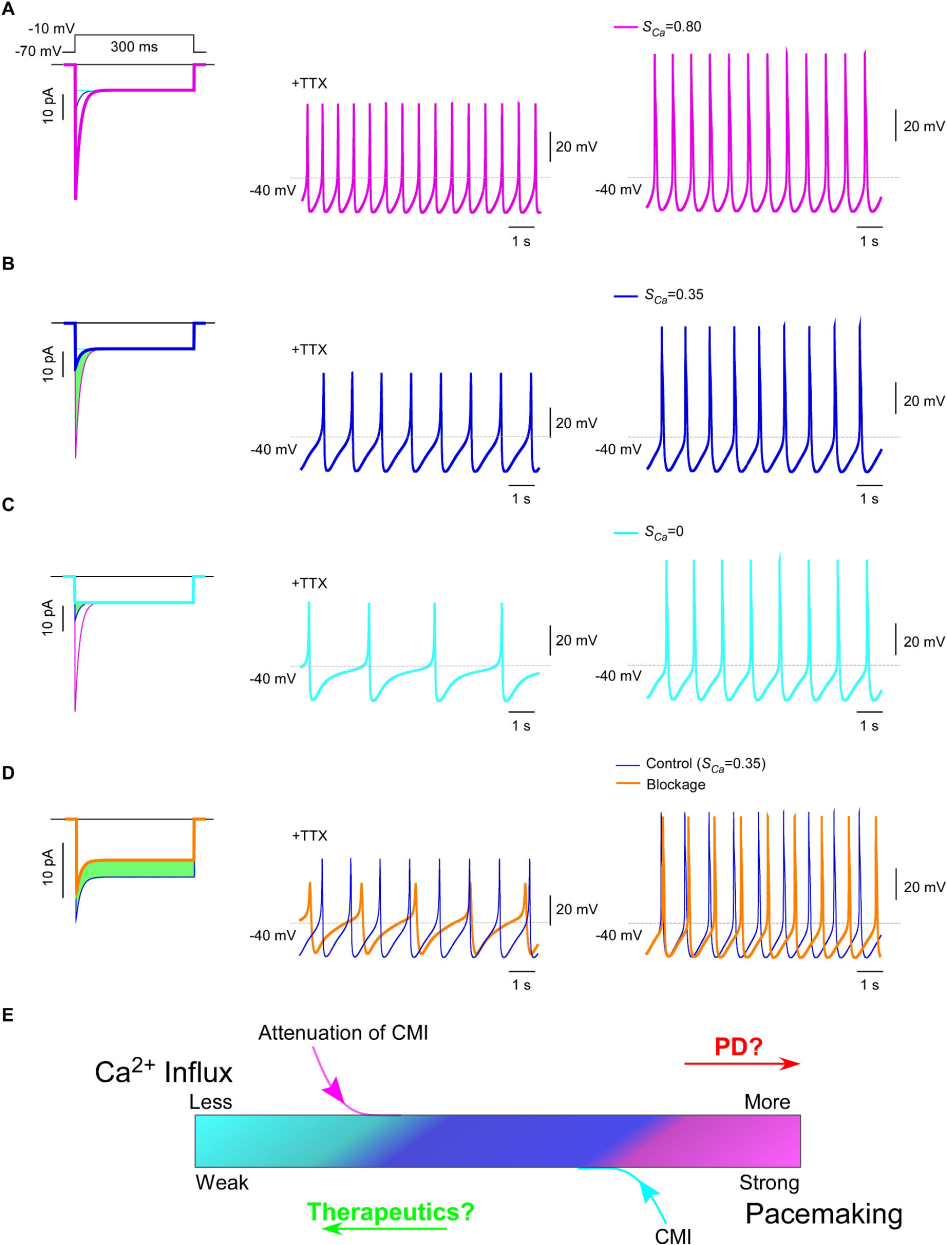
Inhibition of SNc oscillation and pacemaking by CMI. (**A** and **B**) Oscillation and pacemaking of SNc neurons are mediated by Ca_V_1.3, presumably mixed with both short (α_1DS_) and long (α_1DL_) splice variants. Lack of CMI, α_1DS_ (pink) exhibited large *I_peak_* and strong *S_Ca_* (**A**), altogether resulted into more Ca^2+^ influx than α_1DL _(blue), as indicated by the green area (**B**). Side-by-side comparison demonstrated that Ca_V_1.3-dependent oscillation (middle column) and pacemaking (right column) were slowed down, presumably by intrinsic CMI of α_1DL_ in comparison to α_1DS_. (**C**) Effects of ultrastrong CMI (cyan) on SNc neurons. Under the CMI of the maximum potency (left), *I_peak_* was reduced to the lower limit, *i.e.*, the level of *I_300_*, and *S_Ca_* was approaching 0. Accompanied with reduction in Ca^2+^ influx (green area), oscillation (middle) and pacemaking (right) rates were also further reduced compared with α_1DS_ (**A**) or α_1DL_ (**B**). (**D**) Effects of conventional blockage. Conventional inhibition by blockers (orange) attenuated *I_Ca_* (28% reduction) and Ca^2+^ influx (green area) but kept *S_Ca_* unaltered (left), by which oscillation (middle) and pacemaking (right) rates were slowed down compared with the α_1DL_ control (**B**). (**E**) The potential regulatory scheme for Ca_V_1.3-mediated pacemaking in SNc neurons. The actual gating of *I_Ca_* (similar to **B**) could fall into intermediate levels between two extreme conditions of rather weak CMI (apoCaM-bound and capable of strong CDI) (**A**), or ultrastrong CMI (apoCaM-off and abolishment of CDI) (**C**). Hence, both Ca_V_1.3 and pacemaking behaviors are potentially bidirectionally regulated, *e.g.*, apoCaM and apoCaM-binding proteins, to maintain the homeostatic balance of Ca^2+^ influx. Pathological dysregulations of Ca_V_1.3 and the resulted Ca^2+^ imbalance might underlie PD and other diseases, as the rational to develop therapeutic perturbations based on CMI. **DOI:** http://dx.doi.org/10.7554/eLife.21989.020

Ca_V_1.3 plays a pivotal role in subthreshold oscillation and suprathreshold pacemaking in diverse cell types including neurons (Chay and Keizer, 1983; Comunanza et al., 2010), *e.g.*, dopaminergic neurons in the substantia nigra compacta (SNc) (Chan et al., 2007). Pathophysiological linkages of PD or other neurodegenerative diseases with Ca_V_1.3 functions have been evidenced in multiple lines of studies (Guzman et al., 2009; Puopolo et al., 2007), including Ca_V_1.3 antagonists as potential therapeutic interventions (Anekonda and Quinn, 2011; Pasternak et al., 2012; Triggle, 2007). To explore the physiological tuning of CMI and its therapeutic potentials, we constructed a customized model for SNc neuron and Ca_V_1.3 channels using the software Neuron (Carnevale and Hines, 2006). Computational analyses were performed to compare the effects between different levels of CMI attenuations, to simulate the tuning of CMI by multiple factors including endogenous DCTs and alternative splicing, apoCaM fluctuations, and apoCaM buffer proteins such as calpacitin (Gerendasy, 1999; Xia and Storm, 2005). Under CMI of different potency, *I_Ca_* from α_1DS_ (no CMI), α_1DL_ (intermediate CMI), or α_1DS_ with inducible peptide dimerization (ultrastrong CMI), exhibited distinct *I_peak_*, *S_Ca_* and Ca^2+^ influx (Figure 7A—C, left) (green areas indicating the reduction of Ca^2+^ influx). Meanwhile, the time rates of Ca^2+^ oscillation and autonomous spiking in the SNc model were also tuned down to different levels in accordance with *I_Ca_* attenuations or CMI potencies (Figure 7A—C). Experimentally, CMI was compared with other conventional modalities of channel inhibition, for their differences in *I_Ca_* attenuations (Figure 6A, Figure 6—figure supplement 1). In parallel, we also simulated the conventional blockage in the Ca_V_1.3/SNc model (Figure 7D). Similar attenuations in oscillation and pacemaking were observed from the SNc model when the moderate (28%) blockade clearly reduced overall Ca^2+^ influx (green area), supporting the current strategy to develop PD therapeutics based on conventional Ca_V_1.3 antagonists (Gudala et al., 2015; Hurley et al., 2013; Kang et al., 2012; Pasternak et al., 2012).

Collectively, one common rule was deduced for CMI, CDI and other modalities of inhibition: Ca^2+^ influx via Ca_V_1.3 serves as the major index to determine the downstream of oscillation/pacemaking, *i.e.*, reduction of Ca^2+^ influx leads to attenuation of pacemaking whereas enhancement of Ca^2+^ influx causes augmentation of pacemaking. These analyses on the potential roles of CMI together with those on CMI mechanisms (Figure 6C) suggest that multiple molecular processes in CMI, especially those pertaining to apoCaM and the trio complex, are crucial for excitability and signaling of SNc neurons as well as related pathology, such as PD (Hurley et al., 2013; Scharinger et al., 2015) (Figure 7E). In this context, CMI, emerging as one key regulatory mechanism distinct from others, essentially reduces Ca^2+^ influx, local and cytosolic Ca^2+^ concentration, and oscillation and pacemaking, all of which are potentially subject to bidirectional tuning under pathophysiological conditions.

## Discussion

This study focuses on the principle of CMI mediated by multiple carboxyl-tail motifs to compete apoCaM off the channel. Such cooperative competition is applied to rapamycin-inducible inhibition on Ca_V_1.3 channels, unveiling that the channel inhibited by ultrastrong CMI should be functionally equivalent to the channel in end-stage CDI. Furthermore, by acute and cooperative inhibition of Ca^2+^ influx, this unique CMI serves as potential therapeutic interventions, as demonstrated by attenuation of Ca_V_1.3-dependent pacemaking with a computational model of SNc neurons.

### CMI across LTCC family and potential physiological linkages

For LTCCs, gating inhibition by carboxyl-tail motifs (CMI) seems to be a universal mechanism preventing excessive Ca^2+^ influx and intracellular Ca^2+^ overload. In Ca_V_1.1 and Ca_V_1.2 channels, C-termini of α_1_ subunits are subject to proteolytic cleavage and the site of cleavage is located between PCRD and DCRD (Abele and Yang, 2012; Hulme et al., 2005, 2006). Hence, CMI serves as the potential mechanism for endogenous DCRD-containing fragments to attenuate *I_Ca_* by cooperating with PCRD and IQ_V_. For Ca_V_1.3, the possibility of proteolytic cleavage has not yet been fully explored. Instead, DCT is subject to alternative splicing thus generating α_1D_ isoforms in two major categories: with (α_1DL_) or without (α_1DS_ or similar variants) autonomous CMI (Bock et al., 2011; Singh et al., 2008), both broadly expressed in various tissues and organs. Ca_V_1.4 channels have profound CMI due to its strong DCT against apoCaM, as evidenced by weak inactivation and small currents in native cells, altogether allowing sustained Ca^2+^ influx and continuous neural transmission in retinal neurons (Singh et al., 2006) and immune cells (Omilusik et al., 2011). To this end, CMI is a prevalent modality of inhibition across Ca_V_1 family members, awaiting further investigations into the molecular mechanisms and pathophysiology.

### Mechanistic insights into Ca_V_1 gating enlightened by CMI

Based on constitutive and inducible CMI effects, we have gained considerable insights into Ca_V_1 gating. First of all, CMI as an emerging modality of channel inhibition resembles CDI, one of the prominent features of Ca^2+^ channels (Ben-Johny and Yue, 2014). Targeting the mechanisms of CDI, most structural efforts have been directed to channels in Ca^2+^ conditions, evidenced by multiple studies focusing on Ca^2+^/CaM bound with key channel motifs such as IQ or IQ_V_ (Ben Johny et al., 2013; Fallon et al., 2005; Kim et al., 2008; Mori et al., 2008) and NSCaTE (Dick et al., 2008), which have not achieved unequivocal viewpoints toward CDI mechanisms. CMI emphasizes on the importance of apoCaM-bound and apoCaM-off structures in Ca^2+^-free conditions, which represent the states of ‘Activated’ (pre-CMI) and ‘Inhibited’ (post-CMI), promisingly holding the key to understand the core mechanisms that control the gating of Ca^2+^ channels. Functionally, CMI helps clarify the long-standing complication on the overall effects of CDI on the actual Ca^2+^ influx into the cell. Historically, CDI is considered as a negative feedback to autonomously reduce Ca^2+^ entries (Alseikhan et al., 2002; Budde et al., 2002; Liu et al., 2010). On the other hand, stronger CDI is often accompanied with larger amplitude or facilitated activation of *I_Ca_* (Adams et al., 2014; Liu et al., 2016; Singh et al., 2008), altogether raising the question whether Ca^2+^ influx is reduced or enhanced by CDI. Here we provide direct evidence that attenuation of CDI via endogenous factors and mechanisms is essentially a reduction of Ca^2+^ influx, assured by the fixed level of Ca^2+^ current at the late phase of *I_Ca_*, *i.e.*, *I_300_* measured in this work, remaining the same regardless of the changes in *I_peak_* or *S_Ca_*. In this context, CMI (in inverse correlation to CDI strength) is the more direct index of channel inhibition. In contrast, CDI strength itself (*S_Ca_*) could be changed by various mechanisms of action besides [apoCaM] tuning, which may or may not actually reduce Ca^2+^ influx. For instance, channel mutations in congenital diseases, *e.g.*, Timothy syndrome (Splawski et al., 2004), could cause less inactivation in *I_Ca_* and thus elevate the Ca^2+^ entries. Also, small-molecule compounds could produce confounding effects on Ca^2+^ influx by triggering multiple events opposed to each other (Hille, 2001), *e.g.*, roscovintine (Yarotskyy et al., 2010) reportedly damps activation and meanwhile enhances inactivation, raising uncertainties in its actual effects on Ca^2+^ influx.

### Therapeutic potentials of CMI-based interventions for PD and beyond

Ca_V_1.3 acts as the dominant factor underlying subthreshold oscillation and pacemaking activities in SNc dopaminergic neurons, although the detailed mechanisms are not fully elucidated (Chan et al., 2007; Durante et al., 2004; Putzier et al., 2009). It is currently believed that activity-dependent engagement of Ca_V_1 channels elevates mitochondrial oxidant stress and vulnerability of SNc neurons, contributing to disease progression of PD (Guzman et al., 2010). Additional support also comes from clinical evidence that Ca_V_1 blockers for hypertension treatment, *e.g.*, 1,4-dihydropyridines (DHPs) appear to reduce the risk of PD (Pasternak et al., 2012; Ritz et al., 2009). However, concerns are raised on developing PD therapeutics based on DHP or like inhibitors. First of all, it has been argued whether Ca_V_1.3 is indeed a prerequisite for pacemaking and autonomous oscillation, because of pharmacological complications of DHP, including non-specific side effects and discrepancies in dose dependence (Guzman et al., 2009; Putzier et al., 2009). Viral or transgenic delivery of CMI peptides to Ca_V_1.3 channels in neurons could circumvent above problems, in hope to unequivocally confirm the actual role of Ca_V_1.3. Secondly, PD interventions deploying Ca_V_1.3 antagonists could be improved in the following aspects: (1) specificity, for which genetically-encoded CMI-based inhibitors provide the desired specificity intrinsic to Ca_V_1 channels; (2) assurance of Ca^2+^ reduction, for which CMI is mechanistically guaranteed to reduce Ca^2+^ influx; however, certain antagonists may not be able to provide such assurance due to aforementioned reasons.

CMI-based interventions and therapeutics could be beneficial to other diseases in addition to PD. A wide spectrum of mental disorders are closely involved in dysregulations of Ca_V_1 channels, in that disease-linked mutations in α_1_ and β_2_ subunits result into abnormal gating properties (Azizan et al., 2013;Cross-Disorder Group of the Psychiatric Genomics Consortium, 2013;Schizophrenia Working Group of the Psychiatric Genomics Consortium, 2014; Scholl et al., 2013). Encouraged by the results from SNc modeling that the dysregulated Ca_V_1.3, Ca^2+^ influx and pacemaking could be corrected back to normal, we expect CMI and its therapeutic potentials to manifest in diverse pathophysiology including Ca_V_1 channelopathies.

### Historical context and future directions

DCT and DCRD effects have been suggested in a few prior works, *e.g.*, DCT peptides truncated from long variants of Ca_V_1.3 or Ca_V_1.4 to coexpress with the truncated channels lacking the DCT motif (Singh et al., 2008, 2006), mainly to demonstrate the ICDI (inhibition of CDI) effect. Here, to advance the understanding, we clarify that multiple C-terminal motifs cooperatively and acutely compete apoCaM off the channel; and unveil that CMI functionally resembles CDI such that the maximum potency of CMI is preset by end-stage CDI, which also ensures CMI reduces the overall Ca^2+^ influx. Moreover, as one major goal beyond basic biophysics, we achieve CMI-based peptides for Ca_V_1.3 of native forms, including the short splice variant α_1DS_ in this work and potentially also the long variant α_1DL_, another major isoform in native tissues (Yang et al., 2015). In parallel, one study focusing on CaM, the other side of the competition, speculated on CDI mechanisms as a simple CaM-off process based on the data produced with local enrichment of apoCaM (Adams et al., 2014), which, in our view, may have the follow concerns. First, prescreening is needed to control the baseline gating since CaM itself is very well expressed in cells and easily reaches concentrations high enough to diminish the dynamic space to gauge any further change in CDI (Liu et al., 2010). Second, even with above practical issues being handled, any claims about CDI still await further proof with direct evidence based on actual inhibition such as CMI. Third, apoCaM, due to its central position and multifaceted roles in cell signaling (Cheung, 1980; Chin and Means, 2000; Guzman et al., 2010; Hoeflich and Ikura, 2002), is nearly impossible to develop into molecular tools and therapeutics.

To expand CMI-based inhibitors onto in vivo applications, the approach of rapamycin-mediated heterodimerization may not be directly applicable due to potential interferences with normal cell proliferation, growth and survival (MacMillan, 2013). Nevertheless, the proof-of-concept prototypes in this work lay the foundations for further development and optimization, *e.g.*, by way of light-inducible dimerization (Kennedy et al., 2010; Levskaya et al., 2009; Yazawa et al., 2009), to explore the biological ramifications and therapeutic potentials of CMI in diverse settings.

## Materials and methods

### Molecular biology

α_1D_Short_ (denoted as α_1DS_) variants were constructed by introducing a unique XbaI site following the IQ domain. PCRD was cloned from either Ca_V_1.4 α_1F_ (NP005174, GenBank accession number, the same as follows) or Ca_V_1.3 α_1D_ (NM_000720); and DCRD was based on Ca_V_1.4 α_1F_ (NP005174), and CFP/YFP-tagged DCRD constructs were made using a similar process as described previously (Liu et al., 2010). Then other CFP/YFP-tagged constructs were cloned by replacing DCRD with appropriate PCR amplified segments, via unique NotI and XbaI sites, including YFP-PCRD, CFP-PCRD-DCRD, and CFP-CaM. Peptides of IQ_V_ and IQ_V_-PCRD were cloned from Ca_V_1.2 α_1C_ (NM_199460.3) and Ca_V_1.3 α_1D_ (NM_000720), based on preIQ_3_-IQ and preIQ_3_-IQ-PCRD as the templates respectively. YFP-IQ_V_ and YFP-IQ_V_-PCRD were cloned similarly as YFP-PCRD. Segments of PCRD-DCRD, PCRD, DCRD with different glycines (G_0_, G_6_ and G_24_) were PCR-amplified with SpeI and XbaI sites and inserted into aforementioned α_1DS_.BSCaM_IQ_, serving as the apoCaM buffer in this study, was kindly provided by Dr. A. Persechini (Black et al., 2006). Rapamycin-inducible system consisted of FRB, which was based on 93 a.a. rapamycin binding motif of MTOR; and FKBP, which was 108 a.a. human FKBP-12 (AAA58472). Constructs of CFP-PCRD-FRB, YFP-FKBP-DCRD, YFP-FKBP-PCRD and FRB-CFP-ras were made by appropriate design. PCRD segment was amplified by PCR with flanking NotI and EcoRI then cloned directionally via these two unique sites into CFP-FRB_pcDNA4_HisMaxC expressing plasmids. DCRD segment was amplified by PCR with flanking BamHI and EcoRI then cloned directionally via these two unique sites into YFP-FKBP_pcDNA4_HisMaxC expressing plasmids. The fusion of YFP-FKBP-PCRD and FRB-CFP-ras were ligated by overlap extension PCR with flanking KpnI and XbaI then cloned into pcDNA3 expressing plasmids via these two unique sites. To make constructs of PCRD-FRB and FKBP-DCRD, segments from CFP-PCRD-FRB and YFP-FKBP-DCRD respectively were amplified by PCR with flanking KpnI and XbaI then cloned directionally into pcDNA4 vector. For chimeric channel α_1DS_-G_12_-FRB, a linker containing 12 glycine residues was fused with FRB by overlap PCR with flanking SpeI and XbaI and cloned into α_1DS_ containing engineered cloning sites before the stop codon.

### Transfection of cDNA constructs

For whole-cell electrophysiology and confocal fluorescence imaging, HEK293 cells were cultured in 60 mm dishes or 35 mm No. 0 glass-bottom dishes, and constructs were transiently transfected according to an established calcium phosphate protocol (Liu et al., 2010). HEK293 cell line was generously provided by Dr. Zhijie Chang (Tsinghua University). The cell line was free of mycoplasma contamination, checked by PCR with primers 5’- GGCGAATGGGTGAGTAACACG −3’ and 5’- CGGATAACGCTTGCGACCTATG −3’. 5 μg of cDNA encoding the desired α_1D_ subunit, along with 4 μg of rat brain β_2a_ (M80545) and 4 μg of rat brain α_2_δ (NM012919.2) subunits were applied. All of the above cDNA constructs contained a cytomegalovirus promoter. To enhance expression, cDNA for simian virus 40 T antigen (1–2 μg) was also co-transfected with channel constructs. For all the peptides co-transfected, 2 μg of plasmids were added together with channels. Cells were washed with PBS 6–8 hr after transfection and maintained in supplemented DMEM, then incubated for at least 48 hr in a water-saturated 5% CO_2_ incubator at 37°C before electrophysiology experiments and confocal microscopy experiments.

For FRET optical imaging, HEK293 cells cultured on 35 mm No. 0 glass-bottom dishes were transfected with cDNAs (1–5 μg each) by Lipofectamine 2000 (Invitrogen, Waltham, MA). Cells were washed with PBS 4–6 hr after transfection and maintained in supplemented DMEM, then incubated for 24–48 hr in a water-saturated 5% CO_2_ incubator at 37°C before FRET 2-hybrid experiments.

### Whole-cell electrophysiology

Whole-cell recordings of transfected HEK293 cells were performed at room temperature (25°C) using an Axopatch 200B amplifier (Axon Instruments, Sunnyvale, CA). Electrodes were pulled with borosilicate glass capillaries by a programmable puller (P-1000, Sutter Instruments, Novato, CA) and heat-polished by a microforge (MF-830, Narishige, Japan), resulting in 1–3 MΩ resistances, before series resistance compensation of about 70%. The internal solutions contained (in mM): CsMeSO_3_, 135; CsCl_2_, 5; MgCl_2_, 1; MgATP, 4; HEPES, 5; and EGTA, 5, adjusted to 290 mOsm with glucose and pH 7.3 with CsOH. The extracellular solutions contained (in mM): TEA-MeSO_3_, 140; HEPES, 10; CaCl_2_ or BaCl_2_, 10, adjusted to 300 mOsm with glucose and pH 7.3 with TEAOH, all according to the previous reports (Liu et al., 2016, 2010). For run-down experiments, the content of MgATP (Sigma-Aldrich, St. Louis, MO) in the internal solutions was intentionally reduced. Chemical reagents used for blockage experiments (isradipine, Sigma-Aldrich) and drug-inducible experiments (rapamycin, Fisher Scientific, Waltham, MA) were dissolved in DMSO as 10 mM or 1 mM stock solution, stored at −20°C, and then diluted to 10 μM or 1 μM using extracellular Ca^2+^ solution right before experiments. Whole-cell currents were generated from a family of step depolarization (−70 to +50 mV from a holding potential of −70 mV) or a series of repeated step depolarization (−10 mV from a holding potential of −70 mV). Currents were recorded at 2 kHz low-pass filtering of the instrument. Traces were acquired at a minimum repetition interval of 30 s. P/8 leak subtraction was used throughout.

### FRET optical imaging

FRET 2-hybrid imaging experiments were performed with an inverted microscope (Ti-U, Nikon, Japan) with Neo sCMOS camera (Andor Technology, UK). The light source was from the mercury lamp filtered at the appropriate wavelengths for CFP and YFP by the optical filters mounted at the computer-controlled filter wheel (Sutter Instrument) for excitation, subsequently passing the dichroic mirror and the emission filters. Operations and measurements were controlled by the iQ software (Andor Technology). FRET data were acquired and analyzed by an intensity-based two-hybrid assay (3^3^-FRET) as described (Butz et al., 2016; Erickson et al., 2001; Liu et al., 2010), based on

$$FR=1+\frac{FR_{max}-1}{1+\frac{K_{d}}{D_{free}}}$$

where $FR_{max}$represents the maximum FRET ratio FRpertaining to the receptor (YFP), and $D_{free}$denotes the equivalent free donor (CFP-tagged) concentration. By fitting the curve of $FR-D_{free}$with a set of customized Matlab (Mathworks, Natick, MA) codes to iteratively estimate $FR_{max}$and $D_{free}$, effective dissociation equilibrium constant ($K_{d}$) can be achieved for each binding pair to evaluate the (relative) affinity. During imaging, the bath solution was Tyrode’s buffer, containing (in mM): NaCl, 129; KCl, 5; CaCl_2_, 2; MgCl_2_, 1; HEPES, 25; glucose, 30, 300 mOsm, adjusted with glucose and pH 7.3, adjusted with NaOH. Ionomycin (Sigma-Aldrich) was dissolved in DMSO as 1 mM stock solution, stored at −20°C, and diluted to 1 μM using Tyrode’s buffer immediately before applications.

### Confocal fluorescence imaging

Fluorescence images were achieved in HEK293 cells transfected with membrane-localized CFP-tagged FRB and cytosolic FKBP-PCRD/DCRD tagged with YFP. Dynamic translocations were observed with a ZEISS (Germany) Laser Scanning Confocal Microscope (LSM710) through a 100X oil objective and analyzed with ZEN 2009 Light Edition software and Adobe Photoshop CS5 software (Adobe Systems, San Jose, CA).

### Computer simulation

Simulations were performed with NEURON (Carnevale and Hines, 2006), version 7.1. In light of the evidence that both α_1DS_ and α_1DL_ could express in SNc neurons, we simulated Ca_V_1.3 of two extreme settings with distinct inactivation kinetics: one is lack of CDI (ultrastrong CMI) and the other exhibits pronounced CDI representing α_1DS_ (without CMI). CMI effects including the endogenous CMI as in α_1DL_ could then be simulated by adjusting the relative weights of the above two states, resulting into varying (intermediate) levels of *S_Ca_* and *J_Ca_* but with constant *I_300_* throughout. Subsequently, we implemented this new Ca_V_1.3 model and substituted the original L-type Ca^2+^ current mechanism in a published Neuron model of SNc (Chan et al., 2007), where amendments were incorporated to appropriately reproduce oscillation and pacemaking (Guzman et al., 2009; Putzier et al., 2009). To simulate CCB effects, *I_Ca_* and its maximum conductance were decreased by ~30%.

### Data analysis and statistics

Data were analyzed in Matlab and Origin (Origin Software, San Clemente, CA). The standard error of the mean (S.E.M.) and Student’s t-test (unpaired; two-tailed with criteria of significance: *p<0.05; **p<0.01; ***p<0.001) were calculated when applicable.
